# Community-based psychosocial substance use disorder interventions in low-and-middle-income countries: a narrative literature review

**DOI:** 10.1186/s13033-020-00405-3

**Published:** 2020-10-08

**Authors:** Jan Manuel Heijdra Suasnabar, Bethany Hipple Walters

**Affiliations:** 1 Trimbos Institute, Utrecht, The Netherlands; 2 London School of Hygiene and Tropical Medicine, Public Health for Development, London, UK

**Keywords:** Community-based mental health, Mental health, Community psychiatry, Implementation, Substance use disorder, Psychosocial, Alcohol use disorder

## Abstract

**Background:**

Mental health and substance use disorders (SUDs) are the world’s leading cause of years lived with disability; in low-and-middle income countries (LIMCs), the treatment gap for SUDs is at least 75%. LMICs face significant structural, resource, political, and sociocultural barriers to scale-up SUD services in community settings.

**Aim:**

This article aims to identify and describe the different types and characteristics of psychosocial community-based SUD interventions in LMICs, and describe what context-specific factors (policy, resource, sociocultural) may influence such interventions in their design, implementation, and/or outcomes.

**Methods:**

A narrative literature review was conducted to identify and discuss community-based SUD intervention studies from LMICs. Articles were identified via a search for abstracts on the MEDLINE, Academic Search Complete, and PsycINFO databases. A preliminary synthesis of findings was developed, which included a description of the study characteristics (such as setting, intervention, population, target SUD, etc.); thereafter, a thematic analysis was conducted to describe the themes related to the aims of this review.

**Results:**

Fifteen intervention studies were included out of 908 abstracts screened. The characteristics of the included interventions varied considerably. Most of the psychosocial interventions were brief interventions. Approximately two thirds of the interventions were delivered by trained lay healthcare workers. Nearly half of the interventions targeted SUDs in addition to other health priorities (HIV, tuberculosis, intimate partner violence). All of the interventions were implemented in middle income countries (i.e. none in low-income countries). The political, resource, and/or sociocultural factors that influenced the interventions are discussed, although findings were significantly limited across studies.

**Conclusion:**

Despite this review’s limitations, its findings present relevant considerations for future SUD intervention developers, researchers, and decision-makers with regards to planning, implementing and adapting community-based SUD interventions.

Key points A narrative literature review was conducted to identify and describe the different types and characteristics of psychosocial community-based substance use disorder (SUD) interventions in LMICs, as well as the context-specific factors that could have influenced the interventions.

Ten out of the 15 included studies were published in or after 2015, suggesting that there has been a relatively recent increase in efforts to implement and/or study community-based SUD interventions in LMICs.

None of the included studies were conducted in a low-income country. Ten interventions were implemented in Asia, six interventions in Africa, one intervention in Eastern Europe, and two in South America.

Screening and brief intervention (SBI) interventions were proportionately the most commonly implemented. The most common intervention delivery settings were primary health care centers, hospital out-patient settings, neighborhoods and specialized SUD treatment settings.

Most of the reviewed interventions were somehow related to other priority conditions or issues (i.e. integrated interventions), such as sexual behaviors, HIV, tuberculosis (TB), pregnancy, and intimate partner violence (IPV).

Based on the sociocultural factors that influenced some interventions, authors recommended that future interventions should be adapted to improve acceptability by female populations, enhance help-seeking behaviors, and focus more on specific substance use patterns per setting.

## Background

### Global mental health

Quality and accessible mental health services, including substance use disorder services, in low-resource settings are increasingly being recognized as issues of global importance [1–3]. In 2016, the adoption of the United Nations 2030 Sustainable Development Goals (SDGs) pushed for the prioritization of focused action in the field of mental healthcare and substance abuse treatment and prevention [1, 3]. The inclusion of mental health and well-being in the SDGs was motivated by previous calls for action and research findings urging to increase the level of care for mental and substance use disorders [3–5]. Mental health and substance use disorders are the world’s leading cause of years lived with disability (YLDs) and account for 183.9 million disability-adjusted life years (DALYs) [6]. Moreover, substance use disorders can best be understood as primarily mental health conditions; SUDs are often treated by mental health professionals in specialized clinics, hospitals, or out-patient treatment programs.

In low-and middle-income countries (LMICs) the treatment gap for mental disorders is between 76 and 85%, meaning that at least three fourths of people with a mental disorder do not receive treatment [2]. In this context, two key objectives outlined in the WHO Mental Health Action Plan and the UN SDGs are to scale-up community-based mental health services and “strengthen the prevention and treatment of substance abuse” [1, 2].

Low-and-middle income countries (LMICs) are defined as those classified as such by the World Bank [7]. Research has shown that the insufficient quality, availability, and funding for mental and substance use disorder services is a significant barrier for the improvement of mental health systems in LMICs [8, 9]; high-income countries (HICs) spend up to twenty times more than low-income countries on mental health-related prevention, treatment, management, and educational programs [10]. Stakeholders in LMICs believe that four mechanisms are key in order to increase the availability and quality of mental health services: the de-centralization of psychiatric institutions, the implementation of community-based mental health services, the increased availability of adequately trained and supervised mental health staff, and the incorporation of mental health care into primary care settings and general hospitals [11]. Addressing these barriers will be essential in order to narrow the current treatment gap and improve services for mental and substance use disorders in LMICs, and with that reduce the disproportionately high burden of disease due to mental disorders in these settings.

Barriers to the scaling up of substance abuse treatment and prevention efforts in LMICs include a low prioritization of the problem, an inability to detect and treat non-severe substance use disorders at an early stage and in the community, and lower help-seeking by affected populations due to fear of stigma or normative cultural views on substance use [12–17]. These persistent political, structural, and sociocultural issues continue to obstruct efforts to provide effective and equitable care services to the mental health populations most in need of them in LMICs [8, 11, 18, 19].

### Substance use disorders in low-and-middle income countries

People suffering from psychoactive substance use disorders (SUDs) make up a large portion of the underserved mental health population, accounting for at least 20.5% of the 183.9 million DALYs due to mental disorders [6]. This review defines SUDs according to the ICD-10 classification, that is, “mental and behavioral disorders due to psychoactive substance use” including acute intoxication, harmful use, dependence syndrome, withdrawal state, withdrawal state with delirium, psychotic disorder, amnesic syndrome, and residual late-onset psychotic disorder [20]. Psychoactive substances include alcohol, opioids, cannabinoids, sedatives, cocaine, stimulants, hallucinogens, tobacco and volatile solvents [20].

This review focuses on SUDs related to alcohol, cannabis, cocaine, opioid, and amphetamine-type stimulant use, as these have a greater correlation with treatment entry and with comorbidity with other mental disorders [6, 21]. Treatment gap estimates for substance use disorders in LMICs range from 75–95%, with the gap being greater in rural areas [16, 17, 22, 23]. SUD-affected populations in LMICs are two to ten times less likely to receive minimally adequate treatment (defined as the minimum number of visits to treat a disorder) and those with a SUD in these settings are less able to recognize their need for treatment than their HIC counterparts [12, 21]. Further, although *harmful* alcohol use contributes to a greater portion of the alcohol-related harm in LMICs than alcohol *dependence*, many LMICs place a greater focus on detecting and treating alcohol dependence; as a result, harmful/hazardous drinkers are often not identified early on and receive care only when their condition has progressed significantly [24]. Although the same cannot be said with regards to illicit substance use due to a lack of data globally, it has been mentioned that “there is a need to improve our understanding of these basic epidemiological questions about illicit drug use and dependence in order to improve our capacity to respond” [25]. In this context, prioritizing efforts to screen for and treat harmful substance use would present significant benefits to LMICs (i.e. by reducing alcohol-related harm, improving prevention of dependence, and generating previously lacking epidemiological data).

The barriers toward the scale-up of SUD treatment and prevention in LMICs may also be related to how SUDs are formally conceptualized and addressed in these settings. The criminalization of illicit substance use poses unique challenges to the field of public health as it not only limits the range of possible SUD treatment or prevention services in a given setting, but it may also increase the negative health effects and other health risks (e.g. of intimate partner violence, HIV/AIDS, tuberculosis) among drug-using populations [26–30]. Only recently—in 2016, at the United Nations General Assembly Special Session on drugs—have more countries agreed to recognize SUDs as “complex multifactorial health” disorders and begun to shift from a punishment approach towards a public health approach [31]. However, despite this step in the right direction, there remain significant knowledge and implementation gaps in the field of SUD care in LMICs, with actions still largely limited to the dissemination of recommendations and materials [26, 31].

### Substance use disorder treatment and prevention

The ecology of SUDs involves “intrapersonal, inter-personal, and broader systems-level processes” which, from a public health perspective, should each be addressed sufficiently [32, 33]. As mentioned previously, SUDs may range from mild (acute intoxication and harmful use) to severe (dependence and withdrawal syndrome), which means that different intervention types, intervention settings, and intervention intensities are necessary to address the care needs of the entire SUD population [32, 34]. Persons with SUDs may receive treatment through specialized treatment settings such as in-patient detoxification and rehabilitation services, through residential treatment and therapeutic communities, through other mental health services, mutual help organizations, or through outpatient hospital and primary care services; however, there is limited information about the arrangement and functioning of such services in LMICs [13, 15].

An ‘indicated prevention’ approach is recommended to prevent the further development of the disorder in cases of harmful substance use [35], that is, a pattern of use not yet characterized as dependence but which causes physical or mental damage to health [20]. Indicated prevention interventions may be defined as brief “client-centered, goal-oriented” psychosocial interventions with educational and motivational components; they have shown positive results in LMICs when delivered by a non-specialized, primary care workforce in rural and community settings [35]. That said, there is currently insufficient evidence about what intervention models and characteristics may be suitable to address the needs of various SUD populations in LMICs.

Substance use disorder interventions may be pharmacological and/or psychosocial in nature. Pharmacological interventions are normally delivered during the early stages of treatment and involve using antagonist and withdrawal-reducing medications to alleviate withdrawal symptoms and facilitate treatment adherence for physically dependent individuals [32, 36, 37]. Psychosocial SUD interventions are usually face-to-face interventions that focus on the psychological and social aspects of a person’s life [38, 39]. Examples of psychosocial SUD interventions include cognitive-behavioral therapy (CBT), brief interventions (including indicated prevention interventions), interpersonal therapy, self-help groups, family therapy, motivational interviewing, and relapse prevention [34, 38]. Lastly, the sociocultural environments in which interventions are delivered may further affect their effectiveness and implementation (e.g. feasibility and acceptability); studies have shown that culturally-adapting psychosocial SUD interventions may result in improved implementation outcomes by addressing context-specific factors such as stigma, ethnicity and cultural beliefs [40–44]. So far, there is limited evidence about the extent to which SUD interventions are culturally adapted in LMICs or about the facilitators, barriers, or common elements involved in this process.

### Community-based SUD interventions

Community mental health and SUD interventions present significant advantages for mental health service users and systems in LMICs such as reduced costs, greater reach, decreased stigma, and improved quality of life and community functioning for affected populations [32, 45–47]. This review defines community-based mental health interventions as decentralized interventions integrated into primary care settings or general hospitals which are supported by collaborations between a range of stakeholders (formal and informal) and which aim to facilitate independent community functioning and rehabilitation through education, self-care, “goal setting, skills development, and… access to community and environmental resources” [2, 48]. Based on this definition, examples of community-based mental health and SUD interventions may include psychosocial interventions delivered through community facilities, outpatient care settings or mental health centers, “support of people with mental disorders living with their families, supported housing”, assertive community treatment, and pharmacological and harm-reduction interventions [2, 47].

There seems to be a growing number of efforts to implement community-based SUD services in LMICs that are responsive to context-specific needs, possibilities, and barriers [35, 49–51]. Although this topic has not has not yet been systematically investigated, previous individual studies from LMICs have demonstrated that various resource, sociocultural and/or political factors may influence the development, implementation and/or outcomes of community-based SUD interventions [35, 49, 50, 52]. Therefore, an initial attempt to systematically identify and describe the relevant contextual factors that may affect community-based SUD interventions in LMICs seems warranted.

In summary, considering the high prevalence of SUDs in LMICs and the discussed barriers to scale-up SUD services in these settings, there has been an increased demand to implement cost-effective community-based SUD interventions in LMICs. However, the translation into practice is still lacking and research on this matter, especially in relation to psychosocial interventions, remains scarce and limited to high-income settings [2, 32, 35].

### Aims

This review aimed to identify and describe the different types and characteristics of psychosocial community-based SUD interventions in LMICs, and explore what context-specific factors (i.e. policy, resource, sociocultural) may influence such interventions in their design, implementation, and/or outcomes.

## Methods

A narrative literature review was conducted on community-based substance use disorder intervention studies from LMICs. This method was seen as the most suitable research design because such reviews seek to identify and discuss information from various sources about relatively broad topics, topics that have not-yet been sufficiently addressed, and/or topics for which quantitative meta-analysis would not be the suitable [53, 54]. This review followed a systematic data collection process using a defined set of criteria and standardized data extraction tools, which are recommended in order to minimize bias and increase the quality of narrative reviews [54]. For the PRISMA checklist, see Additional file 1: Appendix S4.

The narrative synthesis process for this review was inspired by the Guidance on the Conduct of Narrative Synthesis in Systematic Reviews (CNSSR) [55]. First, a preliminary synthesis of the intervention characteristics was developed and presented through textual descriptions and tabulation. Thereafter, a content and brief thematic analysis was conducted based on the (manually) coded data extracted from the included studies. The data coding process used both deductive and inductive coding techniques [56–58].

### Eligibility criteria

Tables 1 and 2 outline the inclusion and exclusion criteria for this review based on the PICOS model (participants, interventions, comparisons, outcomes, and study design). Since the aim of this review was to identify and describe the *characteristics* of community-based SUD interventions, all study designs were included if they contained a description of the intervention and the process of implementation. No limits were placed on study comparisons or reported outcomes to ensure that the greatest variety of interventions were included.

**Table 1:** Inclusion and exclusion criteria

Inclusion criteria item	Description	Justification
Population	Persons aged 16–65 in LMICs identified as having a psychoactive substance use disorder due to alcohol, cannabis, cocaine, amphetamine-type stimulants, or opiate use, with or without formal diagnosis Substance users aged 16–65 considered to be at-risk for SUDs	Alcohol, cannabis, cocaine, amphetamine-type stimulant and opiate use have a greater correlation with treatment entry and other mental disorders [6, 21] Considering the lack of a sufficiently trained and qualified mental health workforce in LMICs, and considering that a key focus in LMICs is the delivery of mental health services by non-specialized community workers, placing limits on the source of diagnoses or diagnostic standard would limit the relevance of this review and likely reduce the number of eligible studies
Intervention	Community-based treatment and/or indicated prevention interventions with a psychosocial component, such as: Assertive community treatment, cognitive behavioral therapy (CBT), brief interventions, indicated prevention interventions, interpersonal therapy, self-help groups, family therapy, motivational interviewing, and/or relapse prevention Interventions delivered in primary care settings such as primary health care centers or general hospital out-patient services, mental health centers (including day care centers), self-help group settings, social/housing services and vocational support services	The development of SUDs involves complex “intrapersonal, inter-personal and broader systems-level processes” which pharmacological interventions, hospital-based interventions and/or campaigns alone do not sufficiently address [27] The recommended good practice for the treatment of SUDs is a biopsychosocial approach, which considers “genetic, psychological, social, economic, [and] political factors” [26, 28] This study sought to explore the influence of context-specific factors on the development, implementation and outcomes of SUD interventions in LMICs; There is a high treatment gap for SUDs in community and rural settings; A significant portion of the SUD population (i.e. harmful users) receive insufficient or no care, such populations would benefit from lower intensity interventions and indicated prevention interventions which may be delivered in primary care settings and in the community
Comparisons and outcomes	All reported outcomes	To ensure that the greatest variety of interventions were included, which is of relevance for this review as it sought to identify and describe the *characteristics* of community-based SUD interventions
Study design	Qualitative, mixed-methods, and quantitative studies such as descriptive studies, research case studies, pre-post trials, RCTs and evaluation studies	The data relevant to this review’s aims may be obtained from various study designs, placing limits on the types of studies would limit the relevance of this review
Articles	English-language articles published in academic journals that follow a peer-review publication process	The timeline for this review was restricted; broadening the criteria to include grey literature would not be feasible Although this review did not assess risk of bias or evidence quality, it did seek to identify ethically conducted research studies that have gone through a peer-review publication process (required for publication in peer-reviewed journals)
Publication date	2008–2019	Due to the infancy of the field and relatively recent calls for action on matters relating to the focus of this review [2, 31, 59]

**Table 2:** Exclusion Criteria

Exclusion criteria item	Description	Justification
Intervention	Policy or guideline implementation studies Pharmacological interventions without a psychosocial component	The inclusion of these types of interventions would result in data that is too heterogenous for a narrative review Although these types of interventions (policies, guidelines, and pharmacological) may address current priorities in the field, they do so in a different context and through means which are not directly focused on the individual and psychosocial factors involved in the development and treatment of SUDs
Target population	Populations without a SUD such as families, carers, and the general population	For reasons of feasibility and relevance. Although interventions for families and general populations are relevant efforts towards addressing SUDs in LMICs, they are fundamentally and practically different from interventions developed for SUD populations; including them would not be feasible, it would unrealistically broaden this review’s scope, and it would reduce its relevance due to the heterogeneity among interventions

To be included, interventions had to be psychosocial face-to-face interventions delivered in community settings as described in the background section of this manuscript. Interventions had to be targeted at people identified as having a psychoactive substance use disorder due to alcohol, cannabis, cocaine, opiate, or amphetamine-type stimulant use with or without a formal diagnosis; this was to ensure that all interventions for these populations were included regardless of resource limitations or differences in diagnostic materials/standards used [35, 46, 59]. Prevention intervention studies were only included if they fit the criteria for “indicated prevention” according to Kane and Greene, that is, “programs that are targeted towards those who are not only at higher risk for an outcome but who have signs and symptoms… of the outcome itself [i.e. substance use, acute intoxication, harmful use]” [35]. Interventions targeting vulnerable populations, such as HIV-positive populations, were only included if these populations (or a sub-set thereof) also had a SUD and if the interventions also targeted the SUD. As this is an exploratory review, no limits were placed on reported outcomes and no risk of bias assessment was conducted.

### Data collection

Articles were obtained via a search for abstracts on the MEDLINE, Academic Search Complete and PsycINFO databases using the EBSCOHOST online repository [60–62]. The search terms used were based on this study’s inclusion criteria and the Medical Subject Headings (MeSH) *substance*-*related disorders, community health services, primary health care, psychiatric rehabilitation* and *community psychiatry*. In addition, the search string used for this review was informed by terms used in previous reviews relevant to this research topic [35, 42, 63–66]. In summary, the search string used in this review covered four key concepts for which related terms were used: (1) psychoactive substance use disorders, (2) psychosocial intervention, (3) community-based, and 4) low-and middle-income country. Additional file 1: Appendix S1 outlines the search string used for this review and special limiters applied.

After duplicates were removed, all abstracts were screened using a screener sheet (Additional file 1: Appendix S2) that was based on this review’s inclusion criteria to determine if articles may be eligible for inclusion. When there were uncertainties about the eligibility of articles, these were discussed with and reviewed by a second reviewer (BJHW, the second author of this manuscript).

### Data coding and analysis

A data capture sheet (Additional file 1: Appendix S3) was developed based on the screener sheet and previously developed materials [67–69] to further inform inclusion decisions and ensure the systematic collection of relevant data. Thus, the data capture sheet served as the coding framework for this review and facilitated the systematic identification of the main study characteristics.

The pre-defined topics used for deductive coding were those that focused on what/how policy, resource, or sociocultural factors influenced interventions in their design, implementation, and/or outcomes [55, 56]. Context-specific barriers and facilitators were initially defined as the sociocultural, political or resource factors that were reported by the authors to have had an impact on the interventions’ design, implementation and/or outcomes. However, early in the coding process it became apparent that the authors’ discussions of these factors in the context of barriers and/or facilitators were significantly limited across studies. Therefore, to allow for some description and analysis of these contextual factors, the data extraction approach was expanded to include *any* discussion of political, resource, sociocultural and/or implementation factors that may have played a role, either explicitly or implicitly, in the rationale for, planning, delivery, and/or outcomes of the interventions (see Additional file 1: Appendix S3). Lastly, any additional or emerging themes from the extracted data were identified and discussed [56].

## Results

### Included articles

**Fig. 1:**
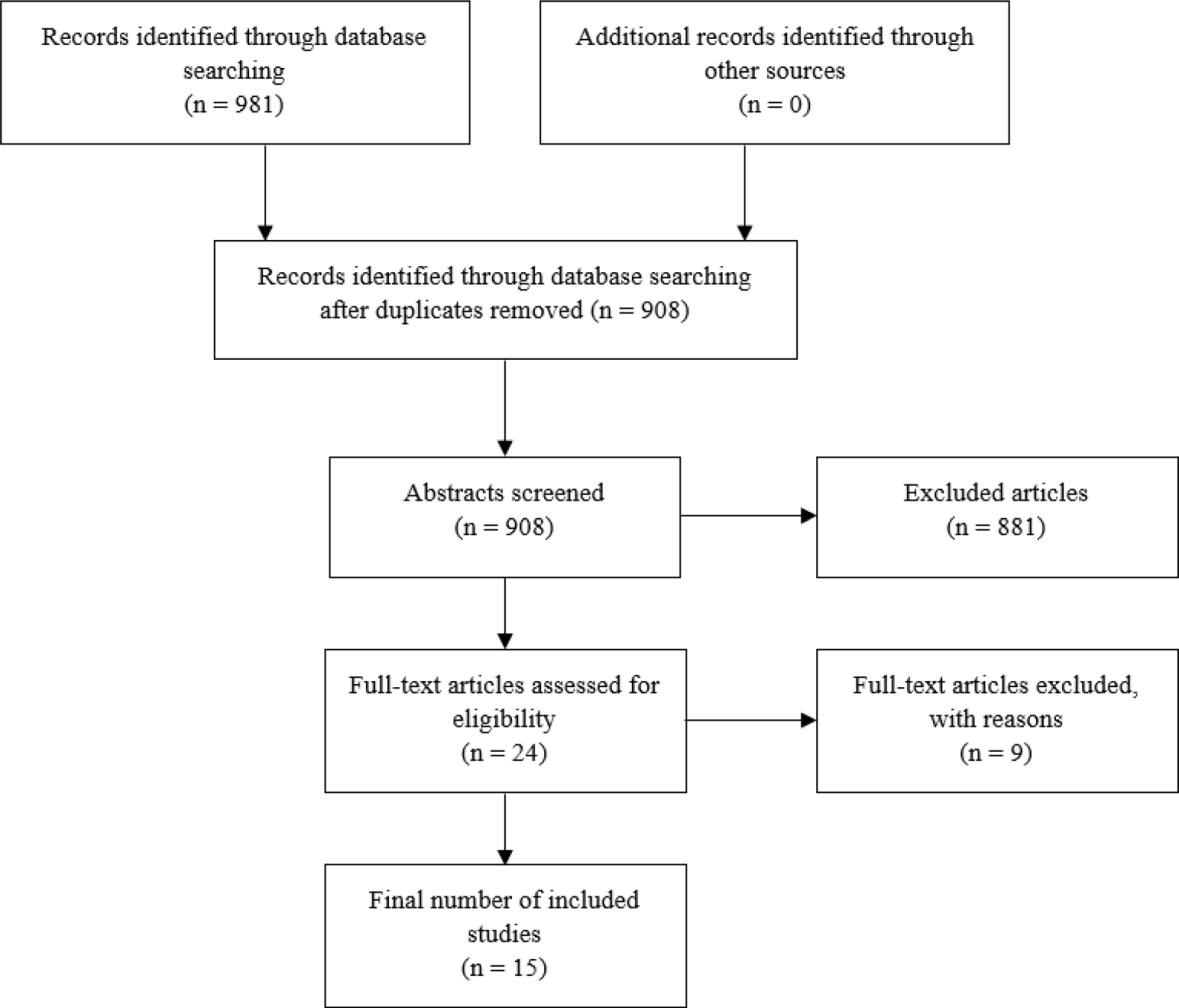
Screening and selection of eligible studies

Figure 1 shows the article selection process of this review according to PRISMA guidelines. Out of 908 abstracts screened, 24 full-text articles were assessed for eligibility; out of these 24, nine articles were cross-checked for eligibility by a second reviewer and eventually excluded. Finally, a total of 15 articles were included for this review. Ten out of the 15 included studies were published in or after 2015, suggesting that there has been a relatively recent increase in efforts to implement and/or study community-based SUD interventions in LMICs. Table 3 presents the key characteristics of the included studies, and Table 4 a description of the interventions and a summary of their findings. The italicized text in Tables 3, 4, and 5 are direct quotations that have been extracted from the included studies.

**Table 3:** Characteristics of included studies

References	Setting	Study design and objectives	Target population/s and condition	Intervention objectives
Almeida do Carmo et al. [87]	Sao Paulo, Brazil	Cross-sectional retrospective to evaluate the effects of a recovery housing and social reintegration program for people recovering from substance dependence	69 persons ages ≥ 18 in recovery from substance dependence, abstinent after discharge from detoxification (alcohol, crack cocaine, marijuana)	Reintegration into society by helping users enter employment, achieve autonomy, remain abstinent and adhere to treatment
Assanangkornchai et al. [86]	Four district hospitals and four healthcare centers in two provinces in Southern Thailand	RCT to *assess the effectiveness of the WHO ASSIST-BI* [78] *procedure compared with ASSIST*-*screening followed by simple advice (SA) in primary care in low*-*population areas*	236 persons ages ≥ 16 identified as problem or risky substance users (alcohol, amphetamine-type substances, cannabis, cocaine, hallucinogens, inhalants, opioids, sedatives and other substances)	Improve identification of substance misuse and provide support for users to understand their risky SU and develop abstinence strategies
Humeniuk et al. [71]	Australia: walk-in sexually transmitted disease clinic. Brazil: 30 primary health care (PHC) units, two health centers and one out-patient setting India: community health centers in Shadipur. United States: community clinic	RCT to *evaluate the effectiveness of a BI* [brief intervention] *for illicit drugs [cannabis, cocaine, amphetamine*-*type stimulants (ATS) and opioids] in PHC clients; determine whether a BI targeted at one substance would increase use of another substance, evaluate whether the general severity of substance involvement affects the response to a BI*	731 persons who scored between 4 and 26 on the ASSIST (moderate-risk range) for cannabis, cocaine, ATS or opioids	Reduce risky substance use (SU) in PHC clients using the WHO ASSIST and its linked brief intervention
Kane et al. [70]	*Three high*-*density, low*-*resource* areas in Lusaka, Zambia	RCT protocol (trial completed). *The primary aims of the trial are to evaluate the effectiveness of the adapted CETA intervention on (a) reducing and preventing women’s experience of* intimate partner violence *(IPV) and (b) reducing male partner’s hazardous alcohol use*	*Hazardous alcohol use and intimate partner violence. Family ‘units’ consisting of three individuals: an adult woman, her male husband or partne*r (who must be a hazardous drinker according to AUDIT scores), ages ≥ 18, *and one of her children (male or female, ages 8*–*17)*	CETA: an adaptable mental health intervention that targets cognitive and behavior change through a variety of intervention components. CETA was specifically adapted in this intervention to be delivered in group settings and to *include a CBT*-*based substance use (SU) reduction element*
Lancaster et al. [76], data also extracted from sister article Miller et al. [77]	*Kyiv, Ukraine (one community site), Thai Nguyen, Vietnam (two district health center sites), and Jakarta, Indonesia (one hospital site)*	Two-arm RCT *designed to determine the feasibility, barriers and uptake of an integrated intervention combining health systems navigation and psychosocial counselling for the early engagement* and adherence *of antiretroviral therapy (ART) and* medication-assisted treatment for substance use *(MAT) for* people who inject drugs (PWID) *living with HIV*	*People who inject drugs (PWID)* more than *12 times per 3* *months (n* = *502), who were HIV*-*positive (viral load of 1000 copies) and their non*-*infected injection partners (n* = *806) were recruited as network units.* Ages 18–60 Conditions: intravenous substance use and HIV	Harm reduction, improved retention and adherence to SU treatment and HIV care, psychosocial counselling*, and referral for ART at any CD4 count*
L’Engle et al. [75]	Three health drop-in centers in Mombasa, Kenya	*RCT to assess whether a brief alcohol intervention leads to reduced alcohol use and sexually transmitted infection (STI)/HIV incidence and related sexual risk behaviors among moderate drinking female sex workers*	Population: Female sex workers of ages ≥ 18 with hazardous drinking (AUDIT score 7–19) Conditions: Alcohol use disorder and STIs	Brief intervention based on WHO Brief Intervention for Alcohol Use. The main objective was to facilitate change/reduction in drinking and risky sexual behaviors
Nadkarni et al. [82]	Eight primary health centers in Goa, India	To study and describe the development of the Counselling for Alcohol Problems (CAP) brief intervention Methods: Three steps are described—*(i) identifying potential treatment strategies; (ii) developing a theoretical framework for the treatment; and (iii) evaluating the acceptability and feasibility of the treatment (through a pilot RCT comparing CAP with enhanced usual care (EUC))*	Males ages ≥ 18 *who had a clinical diagnosis of AUD from a mental health professional or who scored 12*+ *on the Alcohol Use Disorders Identification Test (AUDIT)*	Reduce harmful drinking behaviors through CAP delivery in primary care services by trained non-professionals
Nadkarni et al. [73]	Ten primary health centers in Goa, India	Single-blind individually randomized trial comparing counselling for alcohol problems (CAP) plus enhanced usual care (EUC) versus EUC only	Alcohol dependent males (AUDIT score of 20 or above) 18–64 years old	Investigate the feasibility and cost-effectiveness *of: identifying and recruiting men with probable AD [*alcohol dependence] *in primary care; delivering a brief treatment for AD by lay counsellors in primary care* CAP intervention was used to treat alcohol dependence in primary care
Noknoy et al. [72]	Eight primary care units (PCU) in rural Northeast (n = 7) and central (n =1) Thailand	RCT to determine the *effectiveness of Motivational Enhancement Therapy (MET) for hazardous drinkers in PCU settings*	Hazardous drinkers ages ≥ 18 (AUDIT score of 8 or more)	Reduce alcohol consumption among hazardous drinkers in Thailand and harmful drinking behaviors
Pan et al. [88]	Four community-based Methadone Maintenance Treatment (MMT) clinics in Shanghai, China	RCT to determine [1*] whether CBT is effective in improving treatment retention and reducing drug use for opiate*-*dependent Chinese patients in MMT and* [2] *whether CBT is effective in decreasing addiction severity and psychological stress for MMT patients*. Control group were patients receiving MMT alone	Opiate dependent patients according to psychiatrist diagnosis with DSM-IV. Ages 18–65	Cognitive behavioral therapy alongside methadone maintenance treatment to improve treatment adherence and decrease severity of SUD
Papas et al. [83], data also extracted from Papas et al. [43]	HIV outpatient clinic in Eldoret, Kenya	*RCT of a culturally adapted Cognitive*-*Behavioral Therapy (CBT) to reduce alcohol use among HIV*-*infected outpatients*	Persons ages ≥ 18, enrolled as HIV outpatients (receiving or eligible to receive antiretroviral) who satisfy the hazardous or binge drinking criteria *(score* ≥ *3 on the AUDIT*-*C, or* ≥ *6 drinks per occasion at least monthly*	Culturally adapted CBT to achieve abstinence from alcohol and/or encourage approximations to abstinence
Parry et al. [50]	Durban, South Africa. A number of locations *(i.e. streets in residential and industrial areas, and hotspots where drug users are known to frequent, such as shelters and community*-*based organizations)*	Pre-post intervention study, formal evaluation to t*est whether a community*-*level intervention aimed at alcohol and other drugs (AOD) users has an impact on risky AOD use and sexual risk behavior*	Self-reported alcohol and/or drug users ages ≥ 16	Brief, peer-delivered, risk reduction outreach intervention to reduce AOD use and HIV risky behaviors
Peltzer et al. [84]	Forty primary health care facilities in 3 districts in South Africa	RCT *to assess the effectiveness of screening and brief intervention (SBI) for alcohol use disorders among TB patients in public primary care clinics.* Intervention group received SBI and control group received *treatment as usual in addition to an alcohol education leaflet*	Harmful drinkers (AUDIT scores 7 and above for women and 8 and above for me) ages ≥ 18, currently in treatment for tuberculosis (primary care)	Screening and brief intervention to reduce alcohol misuse delivered by a clinic lay-counsellor *For early identification of alcohol problems in public primary care the AUDIT and for the brief intervention the WHO brief intervention package for hazardous and harmful drinking was used*
Rotheram-Borus et al. [74]	24 low-income urban neighborhoods bordering Cape Town, South Africa	RCT to investigate the effects of a community-based home visiting maternal health intervention by trained non-professional health workers (mentor mothers)	Low income pregnant women Self-reported drinking during pregnancy	Improve maternal health through a home visiting intervention focused on *general maternal and child health, HIV/tuberculosis, alcohol use, and nutrition*
Xiaolu et al. [89]	*18 local hospitals in Beichuan county, China*	*Cluster randomized study… to determine the prevalence of problem alcohol use among the patients from village hospitals and investigate whether a structured BI for those with identified alcohol problems was effective in reducing their alcohol consumption. Nine intervention hospitals and 9 control hospitals*	Persons ages ≥ 18 scoring 7 or above on the AUDIT. Persons *who have experienced a catastrophic event* (i.e. earthquake)	*‘Brief* *Intervention for Substance Use: manual for use in primary care’ recommended by WHO in 2003*

Italicized text are direct quotations extracted from the included studies

**Table 4:** Descriptions of the interventions and main findings

Reference	Intervention characteristics	Summary of findings
Almeida do Carmo et al. [87]	*Recovery Housing (RH) program consisting of accommodation in a drug*-*free environment and a range of structured interventions to address drug and alcohol misuse,* including abstinence-oriented interventions such as multi-professional case management, monitoring of drug use, pharmacological and psychotherapeutic treatment (motivational interviewing, daily care assistance, social and interpersonal functioning, relapse prevention, mutual help). *A collaboration between the state and private companies provides job opportunities and is monitored by a social worker. Residents are also given the opportunity to return to education. The approach to family reintegration is determined on a case*-*by*-*case basis after evaluation of family ties*	*The most common drug use history was use of alcohol and cocaine (72%), followed by alcohol use alone (16%). 51% completed treatment or were reinserted in society (*formal jobs and return to the family*… and 49% had a recurrence during their stay; of the latter, 47% followed treatment at a rehabilitation or psychiatric clinic, 23.5% continued with outpatient treatment, and 29.5% returned to their families and continued treatment elsewhere. Twenty*-*nine of the 34 cases of recurrence occurred in the first 45* *days of residence in the RH, and five between 150 and 180* *days of residenc*e
Assanangkornchai et al. [86]	*The WHO ASSIST*-*linked brief intervention*—*delivered* (by a project worker) *as per the 10*-*step WHO procedure including a Feedback Report Card and Self*-*Help Strategies Manual (part of ASSIST) used to discuss with the patient the meaning of the score and strategies for reducing or stopping their substance use. The focus of intervention was on the substance that resulted in the highest score on the ASSIST or that was of most concern to the participant. The average duration of the BI sessions was 8.8* *min and 3.9* *min for the control group*	*Significant reductions over time in the specific substance involvement scores (SSIS) and total substance involvement scores (TSIS) for both alcohol and other substance users in the BI and SA groups* (non-significant between-group differences). *There were similar patterns of reduction in both groups over time and between facility types. There was an earlier reduction of scores in the sub*-*district health centers than in the district hospitals and participants from sub*-*district health centers changed from moderate*-*risk to low*-*risk faster and in greater proportions than those from district hospitals. At the end of the study 53.3% and 53.4% of the baseline ‘moderate*-*risk’ users in the BI and SA groups, respectively, had become ‘low*-*risk’. Between*-*group comparisons revealed non*-*significant differences in changes in frequency of use for both alcohol and other substances at three and 6* *month*
Humeniuk et al. [71]	*The Brief Intervention (BI) was designed to be relatively short and easily linked to the results of the 7*-*item ASSIST screening questionnaire score via the use of the ASSIST Feedback Report card comprised a major part of the BI. Participants also took the self*-*help guide developed as part of the ASSIST package. The intervention incorporated motivational interviewing techniques that have been found to reduce client resistance while facilitating behavior change. Each country developed their own culturally appropriate brief intervention based on these principles. Average ASSIST baseline screening required 7.9* *min and the BI 13.8* *min. Screening and BI was delivered by trained* project *staff* (treated as clinic staff for the intervention to appear to be routine care) *and (in Brazil) by clinicians and researchers*	*There was a significant reduction over time for the pooled sample regardless of group, and a significant group x time interaction effect in which the group receiving the BI at baseline (regardless of substance) had significantly lower mean total illicit substance involvement scores at follow*-*up than the control group. Participants receiving the BI in Australia, Brazil and India had significantly reduced total illicit substance involvement scores at follow*-*up compared with control participants. There were also significant differences in interaction effects between the countries. Intervention effects were greatest among Australian participants. India and Brazil had a strong BI effect for cannabis, as did Australia and Brazil for stimulants and India for opioids. Although none of the substance*-*specific interaction effects were significant for the United States, there were significant reductions in both the experimental and control groups at follow*-*up for all substances. In general it appeared that severity of use within the moderate*-*risk range (scores 4*–*26) did not influence the success of the BI*
Kane et al. [70]	*CETA is a transdiagnostic mental health intervention developed for delivery by non*-*professionals in LMIC…based on research of common elements or transdiagnostic treatment approaches used in the USA… but with a focus on being appropriate for training and delivery by non*-*professionals in lower resource settings. CETA is based on evidence*-*based treatments for trauma, anxiety, depression, and behavioral problems*. The main components of CETA in this trial include: *psychoeducation and engagement, anxiety management, behavioral activation, cognitive restructuring, exposure, danger assessment and planning, CBT for SU and relapse prevention and safety planning and violence prevention* CBT for SU component delivered to all men and substance-abusing women: included motivational enhancement and goal setting (particularly those related to SU drivers), and teaching and practicing behavior change and avoidance	The trial was completed in January of 2019 (no results published yet). Results (compared to treatment as usual) will include: change in severity of violence against women scale (SVAWS), change in WHO IPV measures, change in youth victimization scale, change in AUDIT scores, change in ASSIST scores, change in CES-D scores (depression), change in Harvard Trauma Questionnaire scores (PTSD), change in child PTSD symptom scale scores, change in aggression scale, change in GEMS score (gender norms), change in Index of Psychological Abuse (psychological violence), change in hair sample cortisol biomarker Modifications to the protocol after first weeks of implementation: CETA delivery was changed to individual delivery instead of through group settings due to challenges in maintaining attendance to group sessions. This also led to an increase in the sample size to 248 families.
Lancaster et al. [76], data also extracted from sister article Miller et al. [77]	*Index participants in the standard of care group received referrals to existing HIV and MAT clinics where they were given primarily methadone; a standardized harm*-*reduction package… and the WHO package of care for PWID… Intervention group received the standard harm*-*reduction package plus the following interventions: systems navigation to facilitate engagement, retention, and adherence in HIV care and MAT, and to negotiate the logistics and… costs of any required laboratory testing (e.g., tuberculosis testing) and transportation; psychosocial counselling by use of motivational interviewing, problem solving, skills building, and goal setting to facilitate initiation of ART and MAT, and if started, medication adherence; and ART initiation. The primary goal for systems navigation was to address individual*-*level or systems*-*level barriers to ART and MAT enrolment… A minimum of two psychosocial counselling sessions (*Lasting 16–60 min*) focused on ART and MAT adherence… tailored to the participant’s needs. Injection partners in both groups received a standardized harm*-*reduction package with referral for MAT*	*Demographic and clinical characteristics at baseline were similar across the intervention and standard of care groups (mostly male, median age of 35). 402 (80%) index participants reported being ART naive at baseline. Only 109 (22%) index participants reported current MAT use at enrolment.* Group distributions across sites were, on average, 125 to the standard of care group and 42 to intervention group. *Findings showed that the intervention was feasible* (80% retention at 52 weeks*), with good intervention uptake, and led to increased ART use, MAT use, and viral suppression. In Vietnam and Ukraine, the intervention effect was positive for self*-*reported ART initiation, viral suppression, self*-*reported MAT initiation, and mortality, whereas in Indonesia the effect of the intervention appeared to be smaller.* MAT uptake was lower than for ART but still significantly higher among intervention participants. *In Vietnam, 42 (86%) of 49 index participants completed two psychosocial counselling encounters within 4* *weeks compared with 30 (64%) of 47 participants in Ukraine and 15 (50%) of 30 participants in Indonesia. More counselling sessions were done with a support person present at the Vietnam and Ukraine*
L’Engle et al. [75]	*Six (approximately 20* *min long) counselling sessions that took place monthly for 6* *months. Nurse counsellors were trained in motivational interviewing techniques and provided the intervention in one*-*on*-*one sessions. Intervention contained elements from stages of change and social cognitive health behavior change theories* and motivational interviewing. Specific (example) goals included: *identification and discussion of risks and consequences from drinking, soliciting participants’ commitment to reduce drinking, identifying the goal of reduced drinking or abstinence, developing a habit*-*breaking plan, discussing high*-*risk situations and coping strategies, and providing feedback and encouragement*	*Nearly* 75% *of participants completed all 5 post*-*enrolment counselling sessions: 292 alcohol intervention participants (71.4%) and 296 nutrition control participants (72.5%). More participants in the intervention group than in the control group reported reduced drinking in the last 30* *days at 6*-*month and 12*-*month follow*-*up visits* (including frequency of drinking alcohol, overall binge drinking, binge drinking with paying clients, and binge drinking with non-paying partners—all statistically significant)*. Women in the intervention group had less than one third of the odds of reporting higher levels of drinking than women in the control group. No between*-*group differences on laboratory*-*confirmed STIs including HIV were detected. The odds of self*-*reported sexual violence from clients was significantly lower among intervention than control participants at both 6 and 12* *months*
Nadkarni et al. [82]	*Motivational Interviewing (MI) techniques were used across all the sessions to help patients develop and maintain their motivation to change.* Several intervention manuals *were identified in consultation with experts as potential starting points for the development of the CAP manual.* CAP was delivered in 3 phases by trained non-professionals: Phase 1: Problem identification with the counsellor using *assessments and personalized feedback. Generating a change and action plan that summarized* the patient’s drinking-related problems and behaviors, and what steps he would take to achieve his behavior-change goals Phase 2: *Helping the patient develop thinking and behavioral skills and techniques* (e.g. drink refusal among others) Phase 3: Learning to *manage potential or actual relapses using these thinking and behavioral skills and techniques* *CAP was delivered in 1 to 4 sessions* at *the patient’s home or the PHC*	*Twenty*-*seven men were assigned to CAP and 26 to EUC. Forty*-*seven (88.7%) participants completed the outcome assessment* and there were no statistically significant baseline characteristic differences between the groups (education, age, occupation, marital status, and AUDIT score). *The amount of alcohol consumed in the past 2* *weeks, mean AUDIT score, and alcohol*-*related problems were all lower in the CAP arm compared to the EUC arm, but the between*-*group adjusted mean differences were not statistically significant. There were nonsignificant reductions in outcomes in participants who completed treatment compared with those who dropped out with regard to mean AUDIT, … mean alcohol consumed in past 2* *weeks, and mean SIP (Short Inventory of Problems, a 15*-*item questionnaire that measures physical, social, intrapersonal, impulsive, and interpersonal consequences of alcohol consumption) score. A number of key barriers were encountered and strategies were modified to address these* (see Table 5)
Nadkarni et al. [73]	The CAP intervention was the same one as the one mentioned above and was delivered by 11 of the same lay-counsellors of the trial for harmful drinkers [73]. *Referral to the local secondary or tertiary care de*-*addiction center for medically assisted detoxification consisted of informing the participants about the need for detoxification, providing them with details about de*-*addiction centers and suggesting that they attend (delivered in out*-*and in*-*patient settings in two district hospitals and one tertiary care psychiatry teaching institute, and private sectors)*	*A total of 66 participants were randomized to EUC and 69 to CAP plus EUC.* *There was no significant difference between the arms for (a) proportion with remission at 3* *months and 12* *months; (b) proportion of participants reporting no alcohol consumption in the past 14* *days at 3* *months and 12* *months; and (c) consumption among those who reported any drinking in this period at 3* *months and 12* *months. At 3* *months, greater expectation of usefulness of counselling was associated with dropout from the study; and at 12* *months, older age and greater readiness to change was associated with dropout from the study* (that said, 89.6% of participants were retained at 3 months and 83% at 12 months). *The mean number of sessions completed was 2.4 (SD* = *1.2) and the mean session duration was 45.9* *min (SD 9.6).* Fifty-eight percent of participants achieved a planned discharge There was *a 20% chance of CAP being cost*-*effective at the willingness*-*to*-*pay threshold of $415. However, from a societal perspective, there was a 53% chance of CAP being cost*-*effective*
Noknoy et al. [72]	Motivational Enhancement Therapy delivered by trained primary care nurses; a brief intervention using the Project MATCH MET protocol (Miller et al. 1992). *The intervention was composed of three scheduled sessions, on Day 1, at 2* *weeks and at 6* *weeks lasting approximately 15* *min.* Different techniques were used depending on the stage of behavior change of the patient (i.e. pre-contemplation, contemplation, determination, action, and maintenance), such as feedback, self-motivational statements, resolving ambivalence, readiness to change assessments, personalized action plans, goal setting, and relapse prevention	*Self*-*reported drinks per drinking day, frequency of daily and weekly hazardous drinking and of binge drinking sessions were reduced in the intervention group more than the control group (P* < *0.05 in 9/10 outcomes assessed) at 3 and 6* *months. The groups did not differ at 3 or 6* *months on self*-*reported frequency of being drunk. The incidence of alcohol*-*related consequences in the 6*-*month period was low in both groups. GGT (a biological marker available for evaluation of the severity of current drinking) levels were higher in both the intervention and control groups at 6*-*month follow*-*up than at baseline. However, the mean GGT level… in the intervention group was lower than… in the control group to a statistically significant degree (P*= *0.038)*
Pan et al. [88]	The participants in the CBT group received individual CBT weekly and group CBT monthly in addition to the standard care of MMT treatment for 26 weeks. The CBT was delivered by psychotherapists experienced in providing counselling or psychotherapy services for patients with SUDs and mental health disorders using an adapted intervention manual. The first 6 weeks focused on building *treatment relationships and enhance motivation for MMT* by helping patients understand *their physical, mental health, social function, legal, economic, family, and employment problems associated with their opiate use,* and by promoting commitment to treatment through signed ‘treatment goals-agreements’. Weeks 7–14 *focused on skills training and… management* of triggers *for opiate use,* as well as developing an individualized treatment protocols and receiving progress feedback to further improve the course of treatment. Weeks *15*–*26 focused on managing psychological stress, building a balanced lifestyle, and maintaining abstinence*	*Participants had a higher (*yet non-significant) *retention rate in the CBT group than the control group* at week 26*. The average proportion of opiate*-*negative urine samples in the CBT group was higher than that in the control group at week 12 (p* = *0.02) and week 26 (p* = *0.02). The average days stay in MMT and mean dosage (mg/day) of methadone did not differ significantly between two groups* *In total, 72.5% completed the follow*-*up interview at week 26 (92 were in the CBT group, 82 were in the control group).* The addiction severity index (ASI) scores decreased significantly over time in both groups with non-significant between-group differences. *Analyses… revealed that the CBT groups improved more on employment function at 26* *weeks, and decreased more on stress level at both week 12 and week 26 compared with the control group*
Papas et al. [83], data also extracted from Papas et al. [43]	Six weekly 90-minute group sessions conducted in Kiswahili by Kenyan, trained non-professionals. The intervention was culturally adapted to best suit local beliefs, drinking behaviors, communications, stigma, gender differences, and HIV-positive diagnosis (see Table 5)	There were *42 CBT and 33 usual care* participants*. Of those randomized to CBT, participants attended 93% of the 6 sessions offered. Results … showed that… reductions since baseline were significantly larger in the CBT condition compared to the usual care condition for both percentage of drinking days (PDD) and number of drinks per drinking day (DDD). Cohen’s d effect sizes of reductions since baseline compared between conditions at 30*-*days post*-*treatment were large and at the 90*-*day follow*-*up were moderate. More CBT than control participants reported abstinence at all follow*-*ups. During the treatment phase, CBT participants reported reducing alcohol use at a faster rate than control participants… During the follow*-*up phase, CBT participants maintained reductions while control participants continued to report gradual reductions over time. It is not known whether differences between conditions increased or decreased beyond* 90 days due to the study design*. Independent ratings of CBT integrity among paraprofessionals showed acceptable adherence and skill ratings*
Parry et al. [50]	*Face*-*to*-*face baseline questionnaire with participants* by peer outreach workers*, risk behaviors* were recorded *and a risk*-*reduction plan was developed with each drug user which consisted of intravenous drug use and non*-*intravenous drug use (IDU/NIDU)*-*related risks, sex*-*related risks and HIV testing.* Thereafter, an *intervention session* was *offered to the clients* covering *education about HIV, condom demonstration and the provision of referrals as needed.* Twenty peer outreach workers were recruited, trained, and paid to deliver the intervention. *At follow*-*up* (varying timeframes)*, both the questionnaire and risk*-*reduction plan were discussed again to assess behavior change and revise risk*-*reduction plans.* There was monthly and bi-annual monitoring of *intervention practices… through observations* and performance ratings *by the project coordinators.* Behavior change and benefits of the intervention were self-reported	*There were only statistically significant reductions in* alcohol use between *time 1 and time 2. No significant differences were observed over time for cannabis, cocaine, heroin and Ecstasy use. There was also no significant change in the frequency of substance use. In total, 45.7% of drug users did not report any changes in the number of different substances used, 23.2% increased the number of different substances they used and 31.1% decreased the total number of different substances used over the follow*-*up period* (non-significant)*. Following the intervention, drug users had significantly fewer sex partners* but *there were no significant differences with regard to frequency of sex or use of condoms… In total, 39.1% of the drug users did not report any changes in the number of different substances used during sex, 21.7% increased the number of different substances that they used during sex and 39.1% decreased the total number of different substances used during sex* (only significant reductions in marijuana use during sex)
Peltzer et al. [84]	*The intervention consisted of two sessions* (approximately 20 min)*, the first immediately after alcohol screening and the second within a month thereafter… In the control condition the clinic lay counsellor provided an alcohol education leaflet… The goals for brief counselling were as follows: (1) To identify any alcohol*-*related problems mentioned in the interview, (2) To introduce the sensible drinking leaflet, emphasis the idea of sensible drinking limits, and make sure that patients realize that they are in the risk drinking category, (3) To provide feedback on the relationship between alcohol and TB treatment, (4) To work through the first 3 sections of* a *problem solving manual, (5) To describe drinking diary cards, ((6) To identify a helper, and (7) To plan a follow*-*up counselling session… The Information*-*Motivation*-*Behavioral Skills (IMB) Model was used in the study to guide the alcohol reduction intervention. The IMB model proposes that information about alcohol misuse and methods of reducing and preventing harmful and/or hazardous drinking is a necessary precursor to risk reduction*	*1196 were randomized into 20 control and 20 intervention clinics* (n=*455,* n=*741*, respectively) … *In 75% of the intervention sessions, the lay counsellors implemented at least 6 of the 7 requisite intervention steps… In addition, it was found that in 96% of the cases of brief intervention, only one session was conducted despite having scheduled a follow*-*up session, and in 4% of cases two sessions. There were significant reductions in AUDIT score… over time across treatment groups…* however *the intervention effect on the AUDIT score was statistically not significant. The intervention effect was also not significant for hazardous or harmful drinkers and alcohol dependent drinkers, alcohol dependent drinkers and heavy episodic drinking, while the control group effect was significant for hazardous drinkers… At 6*-*month follow*-*up the intervention group did not significantly differ to the control group in terms of TB treatment cure or completion rate*
Rotheram-Borus et al. [74]	*Home visiting included prenatal and postnatal visits by community health workers (Mentor Mothers) focusing on general maternal and child health, HIV/tuberculosis, alcohol use, and nutrition* The intervention involved mostly education and support covering *key health topics: HIV/TB, prevention of mother to child transmission of HIV, alcohol, mental health, breastfeeding, and malnutrition.* Moreover, the community health workers (CHWs) were trained to promote *skills to facilitate behavior change: goal setting, problem solving, relaxation, assertiveness, and shaping. On average, CHWs made six antenatal visits, five postnatal visits between birth and 2* *months post 18* *months old. After 18* *months, visits only occurred once every birth, and 1.4 visits/month until the children were 6* *months. Sessions lasted 31* *min each on average*	*Intervention membership was not significantly associated with any baseline variables except for an unexpected significant association with the intervention mothers reporting more depression… Having used alcohol during pregnancy was most associated with IPV at baseline and again at 36* *months, as well as continued alcohol use at 18 and 36* *months. Depression at baseline was most associated with concurrent partner violence, continued depression at 18* *months, and more partner violence and less positive emotional health at 36* *months… Positive emotional health was predicted by less alcohol use, less depression and less IPV at 18* *months, less depression at baseline, and by being in the intervention condition… The intervention reduced depression even though initially the mothers in this condition were more depressed than those in the control condition… There also were significant indirect effects of baseline variables on the 36*-*month outcome variables… In addition to its direct effect, alcohol during pregnancy had an indirect effect on alcohol use at 36* *months. Although the intervention reduced alcohol use in pregnancy, drinking resumed post birth*
Xiaolu et al. [89]	*BI used motivational interviewing techniques and incorporated FRAMES (Feedback, Responsibility, Advice, Menu, Empathy and Self*-*efficacy) skills. The intervention lasts 15*–*30* *min and is based on their alcohol use scores from the AUDIT. This approach is targeted toward non*-*dependent drinkers whose drinking may still be harmful.* Intervention was delivered by 60 village hospital staff who were trained in the technique	*Among the 239 problem drinkers, 47 (19.7%) had high risk drinking (AUDIT scores between 7 and 15), and 192 (80.3%) had harmful drinking (AUDIT were above 15). At follow*-*up assessment, compared with the control group, BI group demonstrated significant reductions in AUDIT… and* self-rating anxiety scale *(SAS) scores… and increased in* substance abuse knowledge scale *(SAKS)… and* general well-being schedule *(GWS) scores… controlling for age, education and baseline disequilibrium measurements. Results from separate ANOVA tests showed that there was a time effect… on AUDIT and SAKS in the BI group. The control group showed increase in SAS and SDS scores, and reduction in GWS scores* (all significant)*. Compared with the control group, BI group showed greater reduction on AUDIT and increased on SAKS after intervention.*

Italicized text are direct quotations extracted from the included studies

**Table 5:** Contextual factors coded data

Reference	Cultural adaptations made	Capacity building of non-professionals	Policy factors discussed	Resource factors discussed	Sociocultural factors discussed	Implementation barriers/facilitators discussed
Almeida do Carmo et al. [87]	N/A	N/A	This program was a direct result of a government launched initiative in 2013 *Virtually all RH residents were not working and therefore would not have the right to apply for the government benefit* due to local laws	N/A	*Most of the residents asked for help by seeking health professionals, not family members.* Authors mention *that family groups may be protective factors, but can also be an important risk factor for crack use, e.g., because of the shame and stigma that affects family relations* *The reason most frequently cited* for relapses *by subjects* (89%) *was the difficulty of establishing family ties and building a social support network*	N/A
Assanangkornchai et al. [86]	*The Thai version of the ASSIST was used to screen patients attending outpatient clinics held at the health centers. As krathom (mitragynine speciosa, Kroth., a traditionally used plant with sedative properties) and krathom cocktail (a mixture of boiled krathom leaf juice and a cola drink with medicines, such as benzodiazepines, antihistamines and ‘cough syrup’) are substances commonly used in the area, they were included in the Thai ASSIST under the ‘other drugs’ category*	N/A	*During the study period, the Thai Government launched a new initiative… with the target of reducing the number of users by 400,000 in the first year…* Strategies *to achieve this* included: *screening for substance abuse in various settings; various forms of compulsory treatment; and relapse prevention programs.* This initiative could have influenced outcomes	*Most previous* similar *studies have been carried out in developed countries.* Authors claim to *have demonstrated that similar studies can be completed in developing countries despite problems in funding, staffing and transport; skepticism; and competing priorities* *In general practice and emergency services… the difference between 8 and 4* *min… can be the main factor that determines whether or not screening is adopted as a routine procedure*	N/A	N/A
Humeniuk et al. [71]	*Each country developed their own culturally appropriate brief intervention (*no examples given)	N/A	N/A	N/A	N/A	N/A
Kane et al. [70]	N/A	*A 10*-*day in*-*person CETA training was conducted by study authors, followed by weekly small group meetings in which lay counsellors practiced the treatment elements with a local supervisor (before providing CETA to clients). Sixty*-*three lay counsellors (20 male, 43 female) and seven supervisors were trained in 2016. Supervisors completed one pilot treatment group to strengthen their* CETA knowledge and skills and are periodically monitored*. Weekly meetings are… held between each local supervisor and a CETA trainer.*	N/A	N/A	N/A	*After the first few weeks of intervention delivery, multiple participants were missing group sessions due to logistical challenges (e.g. work, funerals), which necessitated CETA providers to conduct separate individual sessions for participants that were absent. It became challenging for providers to keep up with the many in*-*between group sessions they had to conduct, and then also had to repeat material in groups if an individual missed and was not available in*-*between group sessions. Participants also indicated frustration in that they did want to participate but there was no flexibility for tardiness (in Zambia, this may be defined as an hour or more late) or work/family scheduling within groups. The challenges were substantial enough that* the authors *would not recommend group CETA in Lusaka, Zambia (urban area), even if it was found to be clinically effective. Therefore,* they *modified CETA to be individually delivered*
Lancaster et al. [76], data also extracted from sister article Miller et al. [77]	N/A	*Trained outreach workers who were knowledgeable about community dynamics, including geographic areas, settings and organizations frequented by PWID, were selected* to do the recruitment*. Outreach workers were trained on basic methods of rapid assessment procedures to target areas of high drug use* Note: counsellors in this study did have previous counselling experience.	*Local government restrictions on MAT access, such as low numbers of MAT clinics (Indonesia, Ukraine) and substantial travel distance (Vietnam), often complicated MAT initiation and retention* *In all three countries, the number of available MAT clinics is increasing due to changes in health policy, consequently, uptake of MAT services among the enrolled cohort may have increased throughout study follow*-*up.*	*The role of systems navigator can be fulfilled by peers, social workers, counsellors, or clinicians*—*the key feature is that navigators understand the local health*-*care system and are able to facilitate entry and retention in care. The role did not require a high level of education*—*some counsellors in Ukraine and Indonesia did not have bachelor’s degrees. The roles of counsellor and systems navigator are conceptually distinct, but in all three sites, the same people served both roles, reducing the number of personnel necessary to implement the intervention*	*For PWID in Vietnam, low levels of education may serve as a barrier for HIV and substance use treatment… the limited number of females in Indonesia and Vietnam accurately reflects the population of PWID in these countries based on culture and historic precedence. Female PWID often face more stigma and discrimination than their male PWID, which can be an additional barrier for engaging in HIV and substance use treatment. Treatment as prevention interventions, as well as substance use treatment, should address vary education levels and integrate female tailored approaches, where appropriate* *Injection network size likely reflects the social norms related to injection behavior in each area… (dense networks are associated with injection practices that increase the risk for HIV transmission)… The larger networks in Ukraine may arise because of the uncertainty of the drug sources and the culture of home*-*made drug preparation*	N/A
L’Engle et al. [75]	*To ensure cultural relevance for FSW in Kenya, focus groups to inform intervention adaptation were held with FSW. Intervention adaptations included incorporating a ladder image to assess motivation and readiness to change because the original ruler image was not understood by less literate FSW and development of visuals depicting real*-*life situations of FSW such as fighting while intoxicated and not drinking while pregnant. Focus group participants also were asked about drinking patterns of FSW and for suggestions they had to reduce risky drinking before engaging in sexual activity; these ideas were incorporated into the intervention visuals and mentioned by counsellors as examples of methods FSW could use to reduce their drinking*	*Nurse counsellors were trained in motivational interviewing techniques and provided the intervention in one*-*on*-*one sessions lasting 20* *min on average. Quality assurance of intervention delivery was provided monthly by an alcohol intervention expert through direct observation of counselling sessions, meeting with the nurse counsellors and presentation of cases, and review of data, assessment, and plan notes.*	N/A	N/A	N/A	Authors found that the AUDIT was not effective enough at detecting drinking behavior changes over time. *For example, some items referred to lifetime experiences and thus limited the ability to measure change in alcohol use or could not be easily or consistently answered by participants who had stopped drinking during the study period. Therefore, before unblinding, these end points* were replaced *with items from the behavioral interview asking about drinking behavior over the last 30* *days that were answered by all participants regardless of current drinking status*
Nadkarni et al. [82]	CAP is entirely a culturally adapted intervention which was developed by *(i) identifying potential treatment strategies; (ii) developing a theoretical framework for the treatment; and (iii) evaluating the acceptability, feasibility, and impact of the treatment.* Further, *data from a case series were used to inform several adaptations to enhance the acceptability of CAP to the recipients and feasibility of delivery by lay counsellors of the treatment.* Four previously published intervention manuals were *evaluated by assessing the adequacy of coverage of selected strategies,* their *suitability for use by lay counsellors, and the extent of adaptations needed for the local context* *CAP employs comprehensive and pictorially dominated psychoeducational materials to engage patients in the treatment* since *the vast majority of patients with harmful drinking in primary care are not specifically seeking help for their drinking problem and many patients have limited literacy.*	*128* applicant non-professionals *were selected for interview,* which *involved a structured questionnaire, a brief role play to test for skills such as empathy and questions to evaluate willingness to be part of a team, communication, and interpersonal skills. Following the interview, 31 candidates were invited for and completed the training. Of these,* 19 completed the internship and delivered CAP under supervision. *During the internship, the lay counsellors delivered CAP to patients in PHC and were supervised in groups by experts drawn from the group of local mental health professionals. CAP was iteratively revised based on observations made continuously through* a *case series.* (See Patel et al., 2014; Singla et al., 2014 for further details)	N/A	It was challenging for *lay counsellors to achieve the standards of competence to deliver MI* *The main strength of this study is the structured methodology used to address the challenges inherent to the development, evaluation, and implementation of psychosocial interventions in low resource and culturally diverse contexts, which in turn has led to an intervention which is acceptable to various stakeholders, feasible to deliver, and hence has greater chances of being effective and scalable. If the resulting intervention is found to be cost*-*effective, then this has major implications for alcohol treatments in low resource settings*	*Treatment engagement was hindered as primary care attenders rarely seek health care for their harmful drinking, and patients and family members are not accustomed to receiving “talking treatments” and express a desire for medications to treat the drinking problem; MI stance was not an acceptable approach in a setting where patients expect prescriptive advice from health professionals; Although CAP emphasized family involvement, family members sometimes saw counselling as a “waste of time” or patients were unwilling to involve family members*	*A third of the patients screening positive for harmful drinking were alcohol dependent. Patients often did not have time for the first session (45 to 60* *min) after screening positive for harmful drinking. Dropout rates were high due to practical barriers such as lack of time to attend counselling because of work commitments and inability to travel to the PHC for financial reasons.*
Nadkarni et al. [73]	See above	See above	N/A	N/A	*The low prevalence rate of alcohol dependence in* the *study might be the result of the stigma associated with alcohol dependence which hinders help*-*seeking and could promote socially desirable responses to the screening and outcome tools*	Alcohol dependence (AD) *may require a more intensive psychosocial treatment, and a brief treatment such as the CAP might not be sufficient to deal with the complex cognitive and behavioral processes associated with AD*
Noknoy et al. [72]	N/A	*Nurses had been trained during a single 6*-*h session, which included an introduction to the research project, lecture and practice exercises to assess the severity of alcohol problems, the effect of alcohol on the patient’s health and the effect of alcohol on the family and society*	N/A	*Motivational interviewing is complex, and extensive practice is required to reach advanced levels. The extent and nature of training provided in this study are clearly sub*-*optimal by current international standards*	The *overall increase in GGT levels at* week 6 *may be because baseline data were collected immediately after ‘Kao Pansaa’, a 3*-*month period of Buddhist retreat during which it is customary for people to avoid wrongdoing, including limiting their alcohol drinking. After this period, normal drinking patterns are usually resumed… the increased mean levels of GGT are in contrast to the reduced alcohol consumption that was self*-*reported… this suggests that the use of self*-*reported data is still liable to social desirability bias. Any such problem may be exacerbated in Thailand where there is a cultural desire to please*	*Fidelity to motivational interviewing was not assessed, so it is unknown to what extent the intervention*—*as delivered*—*represents an optimal and valid test of that particular type of brief intervention* *Because of the small number of women in* the *study, the effect of gender on outcomes* could not be determined.
Pan et al. [88]	Translation of measurement tools/questionnaires to Chinese	*Counsellors received training for the study in a 3*-*day didactic and interactive seminar. The competence of CBT counselling was rated with the validated rating system after training*	N/A	N/A	N/A	*The frequency of collecting urine samples (once every 2* *weeks) may not be sufficient to detect all likely incidents of drug use* *More time may be needed for MMT patients to incorporate the skills learned in CBT and to make the requisite changes from cognition to behaviors, especially with regard to negative attitude* *The protocol of CBT may need to be revised and adapted in the future to address specific characteristics and factors for improving treatment retention for MMT*
Papas et al. [83], data also extracted from Papas et al. [43]	*Gender stratification* of the CBT groups was… *deemed necessary to avoid reinforcement of the secondary status of women and encourage their open discussion and engagement in treatment…* Another *adaptation included an emphasis on financial cost of drinking: participants reported lack of money as both a reason for drinking and a reason for quitting,…* therefore, *the contradiction between these two lines of thought* was addressed by the counsellors*… CBT exercises were adapted (*through a prior process with a multi-disciplinary panel) *to the local setting, for example, addressing peer pressure to drink at chang’aa dens, and* addressing drinking refusals and disclosure of HIV status during sexual encounters (condom use etc.) *Visual aids, treatment materials, and culturally relevant metaphors* were used to deliver the intervention. The intervention was delivered by Kenyan counsellors to ensure the *integration of cultural values, traditions and beliefs*	*Treatment was delivered by two counsellors with no prior CBT experience… The training of paraprofessionals to deliver group CBT* was chosen *in an effort to both accommodate local levels of counselling resources as well as increase the potential for rollout should CBT be shown to be effective.* The *counsellor training was about 175*–*300* *h.*	N/A	Participants were reimbursed their transport costs for all appointments and *frequent phone and text appointment reminders to enhance retention. Because of low attendance in some pilot groups, staff also began to transport willing participants to the first CBT session only to boost treatment engagement in the randomized trial*	CBT group sessions *were closed and gender*-*stratified due to issues of stigma and the consecutive building of knowledge across sessions.* Moreover, counsellors were initially intended to be HIV positive to increase trust and because of HIV stigma, however, this was not feasible due to the inability to sufficiently train HIV-positive counsellors	N/A
Parry, Carney, & Williams [50]	The intervention model was *based on a local adaptation of the World Health Organization’s Training guide for HIV prevention outreach to injecting drug users…* which *lessened the focus on injection drug use*-*related behaviors and increased the focus on substance*-*related sexual HIV*-*risk behavior. In addition, the adapted manual emphasized drugs commonly used in South Africa.*	N/A	N/A	*In South Africa, attempts to bring HIV prevention, treatment and care interventions into services for drug users… have been associated* financial, demographic, and awareness barriers*… it is feasible and acceptable to promote such… services with substance users, but more intensive interventions might be needed to have a substantial impact on substance use and substance use*-*related HIV risk behaviors*	Authors claim that it is *likely that* their *choice of peer outreach workers of similar age and race to most of the drug*-*using study participants as well as their empathetic communication and perseverance played a role in facilitating some of the behavior changes that were observed.*	*The study did not include a control group* so it *is not possible to determine if the changes* observed were *a result of the risk*-*reduction counselling* or *other interventions* that may have *occurred spontaneously. The sample size was also limited due to the intensity of the outreach and follow*-*up activities and thus it is possible that real changes over time were not detected. Furthermore, drug users themselves self*-*reported their substance use, and no biological tests were conducted. Although the study aimed to target both IDUs and NIDUs, IDUs were not reached in this study*
Peltzer et al. [84]	The AUDIT was translated into Tsonga, Northern Sotho, Venda, Afrikaans, Xhosa, Zulu and Tswana	*All lay counsellors and up to four nurses per study clinic who were suitable to deliver the brief counselling intervention received formal training… and supervision prior to the start of the study* *The training… comprised of four elements: orientation to the relevant practice, standardized power point presentation, recorded simulated consultations with trained actors and on*-*going clinical supervision by experienced HSRC staff. Counsellors* were *assessed for adherence to the brief counselling protocol in addition to their behavior and skills*	N/A	N/A	N/A	N/A
Rotheram-Borus et al. [74]	N/A	*Low*-*income women were recruited, trained, and certified to serve as interviewers* (recruitment phase) *… Supervisors monitored and gave feedback on the data quality weekly* *Community health workers (CHWs) were trained for* 1 *month in cognitive*-*behavioral change strategies, role*-*playing, and they also watched videotapes of common situations they might face,* they also received monthly refresher trainings. In total, CHWs were trained for two months and about half of the trainees were eliminated because they did not obtain certification. *CHWs were trained to provide and apply health information about general maternal and child health, HIV/ TB, alcohol use, and nutrition to low*-*income, urban women’s lives… CHWs were certified and supervised biweekly with random observations of home visits*	N/A	N/A	N/A	N/A
Xiaolu et al. [89]	N/A	*A total of 60 staff who worked in the intervention village hospitals were invited to attend training* to deliver the intervention*. The training involved lectures, group activities and role*-*plays with video feedback aimed to ensure trainees understood how to conduct the BI…*	N/A	*The utility of a BI for AUD has been investigated in a variety of settings, including general hospitals, emergency departments and primary healthcare. This study extends that evidence to village hospital settings in remote regions of China with limited medical resources*	N/A	N/A

Italicized text are direct quotations extracted from the included studies

Of the 15 included studies, 13 were randomized controlled trials (RCTs), one was a retrospective cross-sectional study, and one was a pre-post study. Of the 13 RCTs, one was an updated RCT protocol of a trial completed in March of 2019 but for which no final results have yet been published; however, the protocol was included as it includes preliminary data relevant to the scope of this review [70]. Several ‘sister articles’ were identified during the screening and study selection process, that is, different publications which discussed the same intervention; in such cases, data was extracted from both publications when relevant (see ‘Reference’ column in Tables 3 and 4). Furthermore, when authors described relevant information related to an included study (such as the detailed methodology) in other articles, data was also extracted from these articles even if they were not identifiable within the initial 908 search results.

### Study characteristics

This review’s first aim was to identify and describe the characteristics of the included studies; Table 3 provides an overview of these characteristics (i.e. setting, study design and objectives, target population and condition, and intervention objectives).

Nearly every study was carried out in one LMIC, with the exception of two RCTs which were carried out in four and in three countries. In the RCT carried out in four countries [71], two countries were HICs (Australia and the United States). Overall, intervention *settings* ranged from 1–40 sites per country (e.g. districts, cities, neighborhoods, primary healthcare centers) and all but two of the interventions were delivered in more than one site per country. The most common intervention delivery settings were primary health care centers (PHCs) (n = 8 interventions), hospital out-patient settings (n = 3 interventions), neighborhoods (n = 3 interventions) and specialized SUD treatment settings (n = 2 interventions). Moreover, other than one Thai study which was conducted in only rural areas [72], two studies included both rural and urban areas, although this data was not uniformly reported across all studies. In total, 18 SUD treatment or indicated prevention interventions were implemented across 12 countries (excluding Australia and the USA, as they are HICs): five countries (10 interventions) in Asia, three countries (six interventions) in Africa, one country (one intervention) in Eastern Europe, and one country (two interventions) in South America. Per country, the most interventions were implemented in South Africa (n = 3), India (n = 3), Thailand (n = 2), China (n = 2) and Brazil (n = 2); all are currently upper-middle income countries except for India, which is lower-middle income. None of the included studies were conducted in a low-income country.

Table 4 provides a summary of the interventions’ characteristics (including the intervention model/s used, intervention objectives, intervention components, the duration of the interventions, the persons delivering the interventions, and the implementation method) and the findings of the included studies. It should be noted that not all of this data was uniformly reported across the included studies.

Eleven studies targeted both men and women (one of which targeted family units which could include children), two studies, which were set in India, targeted only males [41, 73], and two studies targeted only women (one focused on female sex workers and the other on pregnant women) [74, 75]. Of the 11 studies which targeted both men and women, all of them recruited significantly more men than women with a SUD. However, it is worth noting that about 80% of the women recruited in the three-country study came from Ukraine (the other two countries were Vietnam and Indonesia), which the study authors claimed was reflective of differences in local views (e.g. stigma) and help-seeking behaviors across settings [76, 77]. Population sizes across studies ranged from 69 participants in a single-site cross-sectional study to 1196 participants in the Ukraine-Vietnam-Indonesia RCT.

As per this review’s inclusion criteria, all of the interventions were psychosocial in nature and addressed a range of SUDs. Nearly half of the interventions (n = 9) targeted SUDs alongside other priority conditions or objectives according to the setting, such as HIV and/or tuberculosis (TB), substance-use (SU) related and risky sexual behaviors (n = 2), maternal health (n = 1), response after a natural disaster (n = 1), and intimate partner violence (IPV) (n = 1). Furthermore, nine out of the 18 interventions included in this study addressed alcohol use disorders exclusively; five interventions addressed the use of multiple substances; three interventions targeted intravenous drug use; and one intervention targeted opiate dependent participants. The interventions varied considerably in terms of target SUD and the conditions targeted alongside the SUDs.

Regarding the specific intervention types, screening and brief intervention (SBI) interventions were proportionately the most commonly implemented (n = 10 interventions). All of the brief interventions were based on previously developed guides, protocols, or models (for examples, see 78–81). Although the content, intensities, and lengths of the brief interventions varied, they all shared the core objectives/traits of *problem identification* through discussion of substance use behaviors, *psychoeducation* about SU-related risks and consequences, *goal*-*and*-*motivation*-*setting* to reduce substance use, and *feedback and follow*-*up*. The brief interventions could consist of anywhere between 1–6 sessions and the sessions could last anywhere between 8 and 45 min across studies. In addition to the brief interventions, there were: three CBT-based interventions, one long-term social reintegration and housing-support intervention, and four interventions which essentially consisted of peer outreach, provision of information, and practical and psychosocial support.

### Context-specific factors in community-based SUD care

The second aim of this review was to identify and discuss what/how context-specific factors influenced interventions in their design, implementation and/or outcomes. For this, article data was initially extracted on the *reported barriers and facilitators* related to these factors (see Additional file 1: Appendix S3), however, as mentioned earlier, explicit reports and discussions of these factors in the context of barriers and/or facilitators were significantly limited across studies (this is partly reflected by the general lack of data in Table 5). Therefore, to allow for some description and analysis of these contextual factors, the data extraction approach was expanded to include *any* political, resource, sociocultural and/or implementation factors that may have played a role in the rationale for, planning, delivery, and/or outcomes of the interventions. Accordingly, in this section, we discuss the topics of ‘cultural adaptations made’ and the ‘training and use of non-professionals’, as these topics directly reflect how sociocultural and resource factors influenced the interventions.

### Sociocultural and resource factors

The two most discussed contextual factors were resource and sociocultural factors, which generally appeared to be related. Specifically, the training and use of non-professionals to deliver the interventions (in 11 studies) was often described as a strategy to address local resource limitations and, to a somewhat lesser extent, sociocultural perspectives in different settings. Moreover, the trainings for non-professionals generally consisted of understanding the *disorder/objective in question*, learning patient-centered *communication techniques*, and *practicing acquired skills* [50, 73, 82–84]. These findings may indicate that training non-professionals to deliver community-based SUD interventions in LMICs may be a strategy to address local resource and/or sociocultural barriers.

Regarding the sociocultural factors considered for the planning and delivery of the interventions, the main topics discussed across the included studies were: societal perspectives about gender differences [83], stigma [43], low literacy rates among target populations [41, 85], specific substance use behaviors [50, 83, 85, 86], specific help-seeking behaviors [82]. These factors were discussed in the context of the adaptations that were made to the interventions in order to improve their acceptability by the target populations. For example, in one study, gender stratification of group sessions was “deemed necessary to avoid reinforcement of the secondary status of women and encourage their open discussion and engagement in treatment” [43], while in other studies images were used to assist less literate participants in understanding certain topics [75] or “engage patients in treatment” in a culturally acceptable way [41, 73]. Lastly, nearly all of the studies reported to have translated screening, training and/or intervention materials into local languages. Despite these findings on the sociocultural factors considered for the planning and tailoring of some interventions, this topic was not addressed in most studies, as is reflected by the general lack of data under the ‘cultural adaptations made’ column in Table 5 .

The sociocultural factors that influenced the execution and results of the studies included: stigma against female substance use behaviors [76, 77], limited education/literacy (leading to limited engagement) [41], particular help-seeking behaviors due to fear of stigma [87], local views on punctuality (leading to limited engagement) [83], and the event of a local holiday (which influenced substance use patterns) [72]. Based on these factors, some studies recommended that future interventions should be adapted to improve acceptability by female populations, enhance help-seeking behaviors, and focus more on specific substance use patterns per setting.

The resource-related factors that affected the interventions’ implementation and/or outcomes included: financial and time limitations by the participants, leading to lower participation [41]; the limited capacity of non-professional “counsellors to achieve the standards of competence to deliver” an intervention [41]; the limited capacity to deliver trainings that were in line with “current international standards” [72]; and constraints which impeded significant changes to be detected and/or measured over time [50, 88]. In addition to the use of non-professionals for intervention delivery, few further resource facilitators were discussed across studies.

According to some authors, the characteristics and outcomes of their interventions demonstrated that it was feasible and acceptable to deliver such interventions in LMICs despite possible financial, demographic, and awareness (both societal and from participants) barriers [41, 50, 73, 76, 86, 89]. The few resource-related facilitators to the interventions that were identified involved reimbursing participants for participation and/or transportation costs [83], and task-sharing by non-professionals to adopt multiple yet simple functions [76]. Furthermore, findings from the Thai brief intervention study showed that delivering the brief intervention (which took about 8 min) was as effective in reducing substance use as simple advice (control condition, which took about 4 min); according to the authors, these findings are relevant for future decision-making in low-resource settings where large populations need to be screened in a short amount of time [86]. These examples may reflect (albeit in a limited sense) how SUD interventions in LMICs may be adapted to increase acceptability and be more responsive to the available resources in a given setting.

### Policy-related factors

Only three studies explicitly discussed any influence of policies or governments on the planning, implementation, and/or outcomes of the interventions. In the first study, the Brazilian ‘recovery housing’ intervention was introduced as a government-funded program in response to the “growing public health and social security issue” of substance abuse in the country [87]. Moreover, a policy-related barrier discussed in the ‘recovery housing’ intervention was that most participants were not eligible to receive government benefits because of local law arrangements [87]. Second, during the Thai brief intervention study, “the Thai Government launched a new initiative… [to decrease the number of substance] users by 400,000” in 1 year through a number of strategies, including compulsory treatment [86]; although the authors claim that this initiative likely influenced the study’s favorable outcomes, there was no discussion about the mechanisms by which this could have happened, that is, for example, whether the government initiative was genuinely effective in reducing substance use, or whether it influenced participants’ self-report of substance use to possibly avoid compulsory treatment. Third, authors from the Indonesia-Ukraine-Vietnam study reported that although access to medication assisted treatment (MAT) is still limited in these settings, it has been increasing “due to changes in health policy” in the three countries, therefore, more positive results may be seen in future follow-ups [76]. Lastly, although government funding and/or collaborations were not discussed as facilitators per se, it is worth noting that six studies were at least partly supported by national governmental organizations; the rest received funding from international health/development organizations such as the National Institutes of Health (NIH), the United States Agency for International Development (USAID), the World Health Organization (WHO), and the United States Centers for Disease Control and Prevention (CDC).

## Discussion

This review sought to identify and describe the characteristics of psychosocial community-based SUD interventions in LMICs. Furthermore, this review aimed to discuss the relevant context-specific factors in the field based on the information reported within the included studies. The fact that ten of the fifteen studies were published in or after 2015 (all of them after 2010) suggests a recent rise in efforts to study community-based SUD treatment and indicated prevention interventions in LMICs, which is in-line with recent international calls for action [1–3]. Moreover, the proportionately high number of interventions delivered by non-professionals in this review is in-line with other recent findings in LMICs, strengthening the perspective that utilizing non-professionals is a good practice and convenient strategy to implement community-based SUD services in settings with limited resources [90, 91]. Although these are positive findings for the field of SUD care in LMICs, the low total number of studies identified and the heterogeneity among them still reflect a significantly limited evidence-base on the topic. The heterogeneity across studies is not surprising given this review’s broad inclusion criteria and considering the complexities involved in implementing and studying community-based SUD interventions in different settings. However, researchers and project leaders in the field can and should reinforce the importance of reporting and examining interventions in terms of the sociocultural, resource, and political context factors discussed in this review; this is especially relevant *because* of the variable and mature nature of this field. If the burden of SUDs in LMICs is to be reduced and if SUD services are to become more accessible and more responsive to different settings, then efforts to understand and report the influences of context will need to be intensified. With this greater understanding about how contextual factors may influence the utility, acceptability, adoption, feasibility and/or sustainability of SUD interventions in various settings, project leaders will be better able to design and tailor future SUD interventions in the most appropriate manner.

Most of the included studies were carried out in upper-middle-income countries; no studies were from low-income countries. This finding limits our review’s ability to address its research aims with respect to low-*and*-middle income countries. In addition to the likely greater capacity of upper-middle-income countries to implement community-based SUD interventions, the greater concentration of interventions in these settings could also be due to their seemly greater need for such interventions, as reports have shown that three times more DALYs are lost there due to substance use than in low-income countries [21]. However, low-income countries also more frequently lack adequate “data collection systems on epidemiology and treatment” of SUDs, which limits the credibility of reports on the matter [21]. With that in mind, the absence of studies from low-income countries that met this review’s criteria could also be explained by the likely greater focus on gathering epidemiological data and improving routine data collection systems in low-income countries. Indeed, a number of low-income-country prevalence studies were identified during this review’s article selection process [see 79, 80, 92, 93]. With that in mind, it may be useful to adapt/specify future research questions about SUD interventions or related studies to low-income countries or middle-income countries separately, as it appears to be a premature time to apply this review’s research questions to low-*and* middle-income countries.

To be included in this review, SUD interventions had to be treatment or indicated prevention interventions. Indicated prevention interventions were included because it has been found that a large proportion of at-risk harmful substance users are not sufficiently identified and/or treated [24, 35]. Most of the brief interventions included in this review could be considered to be indicated prevention interventions because they targeted harmful substance users [35, 94]; however, none of them were described as such by the authors. This may be because the field of mental health prevention is still in need of taxonomical clarifications regarding the different types of SUD prevention interventions [95, 96] and/or because ‘harmful substance use’ is itself classified as a sub-type of psychoactive substance use disorders in the ICD-10, which is incompatible with the concept of ‘prevention’. Despite this finding, the brief intervention/indicated prevention model seems to be the current first choice when it comes to implementing previously-reccommended accessible, low intensity and preventative SUD services in LMICs [14, 34, 35].

Most of the interventions in this review were somehow related to other priority conditions or goals in different settings, such as sexual behaviors, HIV, tuberculosis (TB), pregnancy, and intimate partner violence (IPV). Regarding the SUD-related characteristics of these integrated interventions, cognitive-behavioral techniques, motivational enhancement techniques, and (to a lesser extent) peer outreach were reported as key intervention components. Although unexpected, the proportionately high number of integrated programming interventions identified is not surprising as SUDs have been associated with higher rates of IPV, HIV & TB transmission, and risky sexual behaviors [29, 97, 98]. With that in mind, there are clear public health benefits to be gained from integrated SUD interventions, especially in low and middle income settings with large vulnerable populations [29, 99–103]. Integrated SUD interventions may also be particularly suited for LMICs considering the often-limited resources available to reach and treat large underserved populations in these settings [30, 104]. However, the number of integrated interventions identified in this review were too few and their characteristics too different to be able to highlight any further key characteristics among them. This review highlights an evidence-gap in the literature on the characteristics, development, mechanisms, and effects of multi-component, integrated interventions to address SUDs alongside other conditions, which should be addressed in future (possibly less systematic) reviews and studies.

Due to the high variability in the objectives, methods, and reporting styles of the included studies, little can be said about the context-specific factors that influenced the interventions in their rationale, implementation and/or outcomes. This is an interesting finding as previous calls for action and intervention materials have highlighted the importance of considering sociocultural, resource, and political barriers to the implementation of accessible mental and SUD services in LMICs [2, 32, 105]. Therefore, it was not predicted that these factors would be discussed as infrequently and inconsistently as they were across the included studies. This ‘reporting gap’ may be attributable to the generally lacking resources (financial and otherwise) to incorporate more complex research methods (such as implementation research approaches) to evaluate the role of these factors in LMIC settings. Our limited findings on this important theme should encourage funders, researchers and project leaders to prioritize investigating and reporting on the influence of contextual factors in the planning and delivery of SUD interventions.

A notable finding was that about one third of the interventions were adapted to accommodate local literacy rates, sociocultural beliefs on gender differences, help-seeking behaviors, and locally-bound substance use behaviors. This finding may add to the current literature about how community-based SUD interventions can potentially be adapted depending on the context [32, 40, 42]. Moreover, the translation and adaptation of intervention materials, as well as the use of non-professionals, may be useful strategies to address local resource and sociocultural barriers, although it may be challenging to sufficiently train non-professionals in some settings due to time and/or resource limitations [41, 72].

### Limitations

In addition to the limitations that have already been mentioned, some important limitations of this review are that it did not quantitatively analyze the results of the included studies, asses their quality of evidence, or risk of bias. Statistical analyses of results was not within scope of this review, nor would it have been feasible given the heterogeneity of study results. Another limitation may lie in this review’s inclusion/exclusion criteria, as it was often challenging to determine which populations were “at an elevated risk” for SUDs in a way that fit this review’s criteria. Although this limitation was minimized by the assessments of a second, more experienced reviewer, it is still possible that a small number of studies were missed during the article selection process. Finally, as this review looked at only published peer-reviewed articles, publication bias may have affected the number of studies identified.

## Conclusion

This review was a necessary first attempt to explore and describe the current evidence base in the field of community-based SUD care in LMICs. The findings of this review present relevant considerations for the future work of researchers, decision-makers and SUD intervention developers with regards to the planning, implementation and adaptation of community-based SUD interventions in LMICs. Future similar reviews may benefit from focusing on discrete (as opposed to multiple) context-specific factors and review literature beyond intervention studies (such as reports, policy papers, and other non-scientific literature). Authors of future community-based SUD intervention studies will need to prioritize discussing what/how contextual factors played a role in their interventions if this field is to develop further in LMICs. Indeed, sharing this knowledge and lessons learned would improve the utility of future similar reviews and it would enhance our global capacity design and implement community-based SUD interventions that are compatible and responsive to different settings.

## Supplementary information

**Additional file 1.** Appendices 1–4.

## Data Availability

Data sharing is not applicable to this article as no datasets were generated or analysed during the current study.

## Abbreviations

SUD: Substance use disorder
LMIC: Low-and-middle-income country
IPV: Intimate partner violence
BI: Brief intervention
SBI: Screening and brief intervention
DALY: Disability-adjusted life year
YLD: Years lived with disability
HIC: High-income country
CBT: Cognitive behavioral therapy
MAT: Medication assisted treatment

## Declaration

JMHS was employed as a research assistant and project administrator at the Trimbos Institute, the Netherlands, from August 2017 to July 2020. He is currently studying Public Health for Development (MSc) at the London School of Hygiene and Tropical Medicine, as a member of the Class of 2021.

## References

[1] United Nations. THE 2030 AGENDA FOR SUSTAINABLE DEVELOPMENT. 2015. https://sustainabledevelopment.un.org/content/documents/21252030. Agenda for Sustainable Development web.pdf.

[2] World Health Organization (2013). Mental Health Action Plan 2013–2020.

[3] World Health Organization. Mental health included in the UN Sustainable Development Goals. 2016. https://www.who.int/mental_health/SDGs/en/.

[4] Semrau M, Evans-Lacko S, Alem A, Ayuso-Mateos JL, Chisholm D, Gureje O (2015). Strengthening mental health systems in low- and middle-income countries: the Emerald programme. BMC Med..

[5] Prince M, Rahman A, Mayston R, Weobong B, Patel V, Minas H, Prince MJ, Cohen A (2014). Mental health and the global health and development agendas. Global mental health: principles and practice.

[6] Whiteford HA, Degenhardt L, Rehm J, Baxter AJ, Ferrari AJ, Erskine HE (2013). Global burden of disease attributable to mental and substance use disorders: findings from the Global Burden of Disease Study 2010. Lancet..

[7] World Bank. World Bank Country and Lending Groups. 2019. https://datahelpdesk.worldbank.org/knowledgebase/articles/906519-world-bank-country-and-lending-groups.

[8] Eaton J, McCay L, Semrau M, Chatterjee S, Baingana F, Araya R (2011). Scale up of services for mental health in low-income and middle-income countries. Lancet..

[9] Semrau M, Evans-Lacko S, Alem A, Luis Ayuso-Mateos J, Chisholm D, Gureje O (2015). Strengthening mental health systems in low-and middle-income countries: the Emerald programme. BMC Med..

[10] World Health Organization. Mental Health Atlas 2017. Geneva; 2018. http://apps.who.int/iris/bitstream/handle/10665/272735/9789241514019-eng.pdf?ua=1.

[11] Saraceno B, van Ommeren M, Batniji R, Cohen A, Gureje O, Mahoney J (2007). Barriers to improvement of mental health services in low-income and middle-income countries. Lancet.

[12] Degenhardt L, Glantz M, Evans-Lacko S, Sadikova E, Sampson N, Thornicroft G (2017). Estimating treatment coverage for people with substance use disorders: an analysis of data from the World Mental Health Surveys. World Psychiatry..

[13] PREVENTION OF DRUG USE AND TREATMENT OF DRUG USE DISORDERS IN RURAL SETTINGS. Vienna: United Nations Office on Drugs and Crime; 2017. https://www.unodc.org/documents/17-01904_Rural_treatment_ebook.pdf.

[14] Dua T, Barbui C, Clark N, Fleischmann A, Poznyak V, van Ommeren M (2011). Evidence-based guidelines for mental, neurological, and substance use disorders in low-and middle-income countries: summary of WHO recommendations. PLoS Med..

[15] Babor TF, Stenius K. CHAPTER 2. HEALTH SERVICES 2.1 Treatment of substance use disorders within health services. Geneva; 2010. https://www.who.int/substance_abuse/activities/msbatlaschtwo.pdf?ua=1.

[16] Zewdu S, Hanlon C, Fekadu A, Medhin G, Teferra S (2019). Treatment gap, help-seeking, stigma and magnitude of alcohol use disorder in rural Ethiopia. Subst Abus Treat Prev Policy..

[17] Pullen E, Oser C (2014). Barriers to substance abuse treatment in rural and urban communities: a counselor perspective. Subst Use Misuse..

[18] Patel V, Belkin GS, Chockalingam A, Cooper J, Saxena S, Rgen J, et al. Grand challenges: integrating mental health services into priority health care platforms. PLoS Med. 2013;10(5):e1001448. http://www.who.int/3by5/capacity/fs/. 10.1371/journal.pmed.1001448 PMC3666874 23737736

[19] Sarikhani Y, Peivand B, Rafiee M, Kavosi Z, Ravangard R (2020). Key barriers to the provision and utilization of mental health services in low-and middle-income countries: a scope study. Community Ment Health J..

[20] World Health Organization. International Statistical Classification of Diseases and Related Health Problems (ICD) tenth revision, 2nd ed. World Health Organization; 2016. https://icd.who.int/browse10/2016/en.

[21] Fuhr D, Clark N, Poznyak V, Fleischmann A. ATLAS on Substance Use: Resources for the prevention and treatment of substance use disorders. Geneva: World Health Organization; 2010. www.who.int/substance_abuse.

[22] Kohn R, Saxena S, Levav I, Saraceno B (2004). The treatment gap in mental health care. Bull World Health Organ..

[23] Rathod SD, De Silva MJ, Ssebunnya J, Breuer E, Murhar V, Luitel NP, et al. Treatment contact coverage for probable depressive and probable alcohol use disorders in four low- and middle-income country districts: The PRIME Cross-Sectional Community Surveys. 2016; http://r4d.dfid.gov.uk/Project/60851/. 10.1371/journal.pone.0162038 PMC5025033 27632166

[24] Benegal V, Chand PK, Obot IS. Packages of care for alcohol use disorders in low-and middle-income countries. PLoS Med. 2009;6(10). http://www.plosmedicine.org. 10.1371/journal.pmed.1000170 PMC2761617 19859536

[25] Rehm J, Patra J. CHAPTER 1. PSYCHOACTIVE SUBSTANCE USE: EPIDEMIOLOGY AND BURDEN OF DISEASE 1.1 Alcohol. Geneva; 2010. https://www.who.int/substance_abuse/activities/msbatlaschone.pdf?ua=1.

[26] Csete J, Kamarulzaman A, Kazatchkine M, Altice F, Balicki M, Buxton J (2017). Public Health and International Drug Policy: Report of the Johns Hopkins-Lancet Commission on Drug Policy and Health. Lancet..

[27] Keefer P, Loayza N, Soares R. Drug Prohibition and Developing Countries: Uncertain Benefits, Certain Costs. In: INNOCENT BYSTANDERS Developing Countries and the War on Drugs. Washington, DC: The World Bank and Palgrave Macmillan; 2010. http://documents.worldbank.org/curated/en/144831468154466729/pdf/536410PUB0Inno101Official0Use0Only1.pdf.

[28] Haldane V, Cervero-Liceras F, Chuah FL, Ong SE, Murphy G, Sigfrid L (2017). Integrating HIV and substance use services: a systematic review. J Int AIDS Soc..

[29] Gilbert L, Raj A, Hien D, Stockman J, Terlikbayeva A, Wyatt G (2015). Targeting the SAVA (Substance Abuse, Violence and AIDS) Syndemic among Women and Girls: a Global Review of Epidemiology and Integrated Interventions HHS Public Access. J Acquir Immune Defic Syndr.

[30] Li X, He G, Wang H, Bartley Williams A. Consequences of Drug Abuse and HIV/AIDS in China: Recommendations for Integrated Care of HIV-Infected Drug Users. AIDS Patient Care STDS. 2009;23(10). http://www.paper.edu.cn. 10.1089/apc.2009.0015 19799494

[31] Volkow N, Poznyak V, Saxena S, Gerra G (2017). Drug use disorders: impact of a public health rather than a criminal justice approach. World Psychiatry..

[32] United Nations Office on Drugs and Crime. Community Based Treatment Good Practice. Vienna; 2008. http://www.unodc.org/treatnet.

[33] White WL. The mobilization of community resources to support long-term addiction recovery. J Subst Abuse Treat. 2009;36:146–58. http://www.elsevier.com/copyright. 10.1016/j.jsat.2008.10.006 19161895

[34] World Health Organization. mhGAP Intervention Guide Mental Health Gap Action Programme Version 2.0 for mental, neurological and substance use disorders in non-specialized health settings. Geneva; 2016. http://www.who.int. 27786430

[35] Kane JC, Greene MC. Addressing Alcohol and Substance Use Disorders among Refugees: A Desk Review of Intervention Approaches. Geneva: United Nations High Commissioner for Refugees; 2018. https://www.unhcr.org/5c064a8d4.pdf.

[36] Bouza C, Carmen B, Angeles M, Amate A&, María J. Efficacy and safety of naltrexone and acamprosate in the treatment of alcohol dependence: a systematic review. 2004; http://www.nntonline.net. 10.1111/j.1360-0443.2004.00763.x 15200577

[37] Patel V, Araya R, Chatterjee S, Chisholm D, Cohen A, De Silva M, et al. Treatment and prevention of mental disorders in low-income and middle-income countries. Lancet. 2007;3(307):991–1005. http://www.thelancet.com. 10.1016/S0140-6736(07)61240-9 17804058

[38] European Monitoring Centre for Drugs and Drug Addiction. The role of psychosocial interventions in drug treatment. European Monitoring Centre for Drugs and Drug Addiction; 2016. http://www.emcdda.europa.eu/topics/pods/psychosocial-.

[39] Jhanjee S. Evidence based psychosocial interventions in substance use. Indian J Psychol Med. 2014;36(2):112–8. https://www.ncbi.nlm.nih.gov/pubmed/24860208. 10.4103/0253-7176.130960 PMC4031575 24860208

[40] Crunkilton D, Paz J, Boyle D (2005). Culturally Competent Intervention with Families of Latino Youth at Risk for Drug Abuse. J Soc Work Pract Addict..

[41] Nadkarni A, Velleman R, Dabholkar H, Shinde S, Bhat B, Mccambridge J (2015). The systematic development and pilot randomized evaluation of counselling for alcohol problems, a lay counselor-delivered psychological treatment for harmful drinking in primary care in India: the PREMIUM study. Alcohol Clin Exp Res.

[42] Valdez LA, Flores M, Ruiz J, Oren E, Carvajal S, Garcia DO. Gender and Cultural Adaptations for Diversity: A Systematic Review of Alcohol and Substance Abuse Interventions for Latino Males. Subst Use Misuse. 2018;53(10):1608–23. 10.1080/10826084.2017.1417999 29364763

[43] Papas R, Sidle JE, Martino S, Baliddawa JB, Songole R, Omolo OE (2010). Systematic cultural adaptation of cognitive-behavioral therapy to reduce alcohol use among HIV-infected outpatients in western Kenya. AIDS Behav..

[44] Barker J, Hunt G. Natural Recovery: A Cross-Cultural Perspective. In: Carter Sobel L, Klingemann J, editors. Promoting Self-Change From Addictive Behaviors Practical Implications for Policy, Prevention, and Treatment. Zurich: Springer Science + Business Media, LLC; 2007.

[45] Wells K, Miranda MPHJ, Martha L, Bruce MPHM, Alegria N, Wallerstein PH. Bridging Community Intervention and Mental Health Services Research. vol. 161, Am J Psychiatry. 2004. http://ajp.psychiatryonline.org. 10.1176/appi.ajp.161.6.955 15169681

[46] Kakuma R, Minas H, Van Ginneken N, Dal Poz MR, Desiraju K, Morris JE, et al. Series Global Mental Health 5 Human resources for mental health care: current situation and strategies for action. Lancet. 2011;378:1654–63. http://www.who.int. 10.1016/S0140-6736(11)61093-3 22008420

[47] Bertolote J, Epping-Jordan J, Funk M, Prentice T, Saraceno B, Saxena S, et al. World Health Report 2001 Mental Health: New Understanding, New Hope. The World Health Report. Geneva; 2001. https://www.who.int/whr/2001/en/whr01_en.pdf?ua=1.

[48] Kidd SA, Davidson L, McKenzie K (2017). Common Factors in Community Mental Health Intervention: a Scoping Review. Community Ment Health J.

[49] How TB, Morales B, Thirumagal V, Ayub M (2014). Development of a village based treatment model for Afghanistan. Int J Prev Treat Subst Use Disord..

[50] Parry C, Carney T, Williams P (2017). Reducing substance use and risky sexual behaviour among drug users in Durban, South Africa: Assessing the impact of community-level risk-reduction interventions. SHARA-J J Soc Asp HIV/AIDS..

[51] Vorhölter J (2017). Class-based chronicities of suffering and seeking help: comparing addiction treatment programs in Uganda. Cult Med Psychiatry.

[52] Galai N, Sirirojn B, Aramrattana A, Srichan K, Thomson N, Golozar A (2018). A cluster randomized trial of community mobilization to reduce methamphetamine use and HIV risk among youth in Thailand: Design, implementation and results. Soc Sci Med..

[53] Baumeister RF, Leary MR. Writing Narrative Literature Reviews. Rev Gen Psychol. 1997;1(3):311–20. http://endoexperience.com/documents/literature_reviews_researched.pdf.

[54] Ferrari R, Ferrari Milan R. Writing narrative style literature reviews Writing narrative style literature reviews Correspondence to. Eur Med Writ Assoc. 2015;24(4). https://www.researchgate.net/publication/288039333.

[55] Popay J, Arai L, Rodgers M, Britten N. Guidance on the conduct of narrative synthesis in systematic reviews: A product from the ESRC Methods Programme Claims making in direct-to-consumer genetic testing: A policy driven analysis of marketing and media View project Information needs of adolesce. 2006; https://www.researchgate.net/publication/233866356.

[56] Vaismoradi M, Turunen H, Bondas T (2013). Content analysis and thematic analysis: implications for conducting a qualitative descriptive study. Nurs Heal Sci..

[57] Braun V, Clarke V. Using thematic analysis in psychology. Qual Res Psychol. 2006;3(2):77–101. http://eprints.uwe.ac.uk/11735/2/thematic_analysis_revised.

[58] Elo S, Kyngäs H (2008). The qualitative content analysis process. J Adv Nurs.

[59] World Health Organization. Mental Health Gap Action Programme: Scaling up care for mental, neurological, and substance use disorders. Geneva; 2008. https://apps.who.int/iris/bitstream/handle/10665/43809/9789241596206_eng.pdf?sequence=1. 26290926

[60] U.S. National Library of Medicine. PubMed. 2019. https://www.ncbi.nlm.nih.gov/pubmed/.

[61] American Psychological Association. PsycINFO. 2019. https://www.apa.org/pubs/databases/psycinfo/.

[62] EBSCO Industries. EBSCO. 2019. https://www.ebsco.com/.

[63] Rane A, Church S, Bhatia U, Orford J, Velleman R, Nadkarni A. Psychosocial interventions for addiction-affected families in Low and Middle Income Countries: A systematic review. Addictive. Addict Behav. 2017;74:1–8. http://researchonline.lshtm.ac.uk/4651051/. 10.1016/j.addbeh.2017.05.015 28554034

[64] Brooke-Sumner C, Petersen I, Asher L, Mall S, Egbe CO, Lund C (2015). Systematic review of feasibility and acceptability of psychosocial interventions for schizophrenia in low and middle income countries. BMC Psychiatry..

[65] Jiwa A, Kelly L, St Pierre-Hansen N. Healing the community to heal the individual Literature review of aboriginal community-based alcohol and substance abuse programs Clinical Review. Can Fam Physician. 2008;54:1000–1. www.cfp.ca. PMC2464791 18625824

[66] Fiestas F, Ponce J (2012). Eficacia de las comunidades terapéuticas en el tratamiento de problemas por uso de sustancias psicoactivas: una revisión sistemática. Rev Peru Med Exp Salud Publica..

[67] McAuley L, Ramsay C. Cochrane Effective Practice and Organisation of Care Review Group DATA COLLECTION CHECKLIST. Ottawa: Cochrane Effective Practice and Organisation of Care Review Group (EPOC); 2002. https://methods.cochrane.org/sites/methods.cochrane.org.bias/files/public/uploads/EPOC Data Collection Checklist.pdf.

[68] Montgomery P, Underhill K, Gardner F, Operario D, Mayo-Wilson E (2013). The Oxford Implementation Index: A new tool for incorporating implementation data into systematic reviews and meta-analyses. J Clin Epidemiol..

[69] Hipple Walters B, Pena-Rey Lorenzo I, Heijdra J. EU Good Practices in Mental Health evaluation tool. Trimbos Institute and European Comission; 2018.

[70] Kane JC, Skavenski S, Wyk V, Murray SM, Bolton P, Melendez F (2017). Testing the effectiveness of a transdiagnostic treatment approach in reducing violence and alcohol abuse among families in Zambia: study protocol of the Violence and Alcohol Treatment (VATU) trial. Glob Ment Heal..

[71] Humeniuk R, Ali R, Babor T, Lucia M, Souza-Formigoni O, Boerngen De Lacerda R, et al. A randomized controlled trial of a brief intervention for illicit drugs linked to the Alcohol, Smoking and Substance Involvement Screening Test (ASSIST) in clients recruited from primary health-care settings in four countries. Addiction. 2011;1–10. http://www.randomization.com/. 10.1111/j.1360-0443.2011.03740.x 22126102

[72] Noknoy S, Rangsin R, Saengcharnchai P, Tantibhaedhyangkul U, Mccambridge J. RCT of Effectiveness of motivational enhancement therapy delivered by nurses for hazardous drinkers in primary care units in Thailand. Alcohol Alcohol. 2010;45(3):263–70. https://academic.oup.com/alcalc/article-abstract/45/3/263/208772. 10.1093/alcalc/agq013 PMC4394355 20236990

[73] Nadkarni A, Weiss HA, Velleman R, McCambridge J, McDaid D, Park A-L, et al. Feasibility, acceptability and cost-effectiveness of a brief, lay counsellor-delivered psychological treatment for men with alcohol dependence in primary care: an exploratory randomized controlled trial. Addiction. 2019; http://nextgenu.org/course/view.php?id=167#0. 10.1111/add.14630 PMC6563185 30957341

[74] Rotheram-Borus MJ, Tomlinson M, Roux I Le, Stein JA. Alcohol Use, Partner Violence, and Depression: A Cluster Randomized Controlled Trial Among Urban South African Mothers Over 3 Years. Am J Prev Med. 2015;. 10.1016/j.amepre.2015.05.004 PMC4615286 26231855

[75] L’Engle K, Mwarogo P, Kingola N, Sinkele W, Weiner DH. A randomized controlled trial of a brief intervention to reduce alcohol use among female sex workers in Mombasa, Kenya. J Acquir Immune Defic Syndr. 2014;67:446–53. https://oce.ovid.com/article/00126334-201412010-00015/HTML. 10.1097/QAI.0000000000000335 25197826

[76] Lancaster KE, Hoffman IF, Hanscom B, Viet Ha T, Dumchev K, Susami H (2018). Regional differences between people who inject drugs in an HIV prevention trial integrating treatment and prevention (HPTN 074): a baseline analysis. J Int AIDS Soc..

[77] Miller WC, Hoffman IF, Hanscom BS, Ha T V, Dumchev K, Djoerban Z, et al. A scalable, integrated intervention to engage people who inject drugs in HIV care and medication-assisted treatment (HPTN 074): a randomised, controlled phase 3 feasibility and efficacy study. Lancet. 2018;392:747. http://www.thelancet.com. 10.1016/S0140-6736(18)31487-9 PMC6299325 30191830

[78] Humeniuk RE, Henry-Edwards S, Ali RL, Poznyak V, Monteiro M (2010). The ASSIST-linked brief intervention for hazardous and harmful substance use: manual for use in primary care.

[79] Henry-Edwards S, Humeniuk R, Ali R, Monteiro M, Poznyak V. Brief Intervention for Substance Use: A Manual for use in primary care. Geneva; 2003. https://www.who.int/substance_abuse/activities/en/Draft_Brief_Intervention_for_Substance_Use.pdf.

[80] Miller WR, Hester R, Connors G, Rychtarik R, Randall C, Anton R (1995). Motivational enhancement therapy manual: A clinical research guide for therapists treating individuals with alcohol abuse and dependence.

[81] Fisher WA, Fisher JD, Harman J (2003). The information-motivation-behavioral skills model: a general social psychological approach to understanding and promoting health behavior. Soc Psychol Found Heal Illn..

[82] Nadkarni A, Velleman R, Dabholkar H, Shinde S, Bhat B, Mccambridge J (2015). The systematic development and pilot randomized evaluation of counselling for alcohol problems, a lay counselor-delivered psychological treatment for harmful drinking in primary care in India: The PREMIUM Study. Alcohol Clin Exp Res..

[83] Papas RK, Sidle JE, Gakinya BN, Baliddawa JB, Martino S, Mwaniki MM (2011). Treatment outcomes of a Stage 1 cognitive-behavioral trial to reduce alcohol use among HIV-infected outpatients in western Kenya. Addiction..

[84] Peltzer K, Naidoo P, Louw J, Matseke G, Zuma K, Mchunu G (2013). Screening and brief interventions for hazardous and harmful alcohol use among patients with active tuberculosis attending primary public care clinics in South Africa: results from a cluster randomized controlled trial. BMC Public Health..

[85] L’Engle KL, Mwarogo P, Kingola N, Sinkele W, Weiner DH (2014). A randomized controlled trial of a brief intervention to reduce alcohol use among female sex workers in Mombasa. Kenya. J Acquir Immune Defic Syndr..

[86] Assanangkornchai S, Nima P, Mcneil EB, Guy Edwards J (2015). Comparative trial of the WHO ASSIST-linked brief intervention and simple advice for substance abuse in primary care. Asian J Psychiatr..

[87] Carmo D, Motta Palma S, Ribeiro A, Paulino Trevizol A, Brietzke E, Abdalla R (2018). Preliminary results from Brazil’s first recovery housing program. Trends Psychiatry Psychother..

[88] Pan S, Jiang H, Du J, Chen H, Li Z, Ling W, et al. Efficacy of Cognitive Behavioral Therapy on Opiate Use and Retention in Methadone Maintenance Treatment in China: A Randomised Trial. PLoS ONE. 2015;10(6):127598. https://journals.plos.org/plosone/article/file?id=10.1371/journal.pone.0127598&type=printable. 10.1371/journal.pone.0127598 PMC4479610 26107818

[89] Xiaolu R, Wenwen W, Ali R, Xu L, Hong W, Min Z, et al. Feasibility of Studying a Brief Intervention to Help Chinese Villagers with Problem Alcohol Use After an Earthquake. Alcohol Alcohol. 2017;52(4):472–6. https://academic.oup.com/alcalc/article-abstract/52/4/472/3077112. 10.1093/alcalc/agx014 28371811

[90] Barnett ML, Gonzalez A, Miranda J, Chavira DA, Lau AS (2018). Mobilizing community health workers to address mental health disparities for underserved populations: a systematic review. Adm Policy Ment Heal..

[91] Petersen I, Evans-Lacko S, Semrau M, Barry MM, Chisholm D, Gronholm P (2016). Promotion, prevention and protection: interventions at the population- and community-levels for mental, neurological and substance use disorders in low- and middle-income countries. Int J Ment Health Syst..

[92] Ayano G, Assefa D, Haile K, Chaka A, Solomon H, Hagos P, et al. Mental, neurologic, and substance use (MNS) disorders among street homeless people in Ethiopia. Ann Gen Psychiatry. 2017;16:40. https://annals-general-psychiatry.biomedcentral.com/track/pdf/10.1186/s12991-017-0163-1. 10.1186/s12991-017-0163-1 PMC5693477 29176996

[93] Nalwadda O, Rathod SD, Nakku J, Lund C, Prince M, Kigozi F. Alcohol use in a rural district in Uganda: Findings from community-based and facility-based cross-sectional studies. Int J Ment Health Syst. 2018 Apr 3;12. https://library3.webster.edu/login?url=https://search.ebscohost.com/login.aspx?direct=true&db=psyh&AN=2018-14534-001&site=ehost-live. 10.1186/s13033-018-0191-5 PMC5883606 29632551

[94] Renstrom M, Ferri M, Mandil A. Eastern Mediterranean Health Journal La Revue de Santé de la Méditerranée orientale 198 1 World Health Organization. vol. 23. 2017. 10.26719/2017.23.3.198 28493267

[95] O’connell ME, Boat T, Warner KE. Committee on the Prevention of Mental Disorders and Substance Abuse Among Children, Youth, and Young Adults: Research Advances and Promising Interventions. Washington, D.C.: National Academy Press; 2009. http://www.nap.edu.

[96] Lenoue SR, Riggs PD. Substance Abuse Prevention. Child Adolesc Psychiatr Clin N Am. 2016;25(2):297–395. 10.1016/j.chc.2015.11.007. 26980131

[97] Luo X, Duan S, Duan Q, Pu Y, Yang Y, Ding Y, et al. Alcohol Use and Subsequent Sex among HIV-Infected Patients in an Ethnic Minority Area of Yunnan Province. PLoS ONE. 2013;8(4):61660. http://www.plosone.org. 10.1371/journal.pone.0061660 PMC3633954 23626712

[98] Ulibarri MD, Hiller SP, Lozada R, Rangel MG, Stockman JK, Silverman JG (2013). Prevalence and characteristics of abuse experiences and depression symptoms among injection drug-using female sex workers in Mexico. J Environ Public Health..

[99] Moore TM, Stuart GL, Meehan JC, Rhatigan DL, Hellmuth JC, Keen SM. Drug abuse and aggression between intimate partners: a meta-analytic review. Clin Psychol Rev. 2008;28:247–74. http://www.sciencedirect.com. 10.1016/j.cpr.2007.05.003 17604891

[100] Singer M, Clair S. Syndemics and Public Health: Reconceptualizing Disease in Bio-Social Context. vol. 17, Anthropology Quarterly. 2003. 10.1525/maq.2003.17.4.423 14716917

[101] Lönnroth K, Williams BG, Stadlin S, Jaramillo E, Dye C. Alcohol use as a risk factor for tuberculosis—a systematic review. BMC Public Health. 2008;8. 10.1186/1471-2458-8-289 PMC2533327 18702821

[102] Marais BJ, Lönnroth K, Lawn SD, Migliori GB, Mwaba P, Glaziou P, et al. Tuberculosis comorbidity with communicable and non-communicable diseases: Integrating health services and control efforts. Vol. 13, The Lancet Infectious Diseases. Elsevier; 2013. p. 436–48. 10.1016/S1473-3099(13)70015-X 23531392

[103] Singer M (2000). A dose of drugs, a touch of violence, a case of aids: conceptualizing the SAVA syndemic. Free Inq-Spec Issue Gangs, Drugs Violence..

[104] Parcesepe AM, Mugglin C, Nalugoda F, Bernard C, Yunihastuti E, Althoff K (2018). Screening and management of mental health and substance use disorders in HIV treatment settings in low-and middle-income countries within the global IeDEA consortium and the International epidemiology Databases to Evaluate AIDS (IeDEA) Consortium. J Int AIDS Soc..

[105] World Health Organization. mhGAP operations manual Mental Health Gap Action Programme (mhGap). Geneva; 2018. http://apps.who.int/bookorders.

