# Supplementary material for: Community-based psychosocial substance use disorder interventions in low-and-middle-income countries: a narrative literature review
Source: Int J Ment Health Syst. 2020 Oct 8;14:74. doi: 10.1186/s13033-020-00405-3 (PMC7542947; doi:10.1186/s13033-020-00405-3)
Supplement: Supplementary file 1 — Additional file 1. Appendices 1–4. [file 13033_2020_405_MOESM1_ESM.docx]

### Appendix S1: Key concepts and search string used

1. *“Psychoactive substance use disorder” OR*

“hazardous alcohol use” OR SUD OR AUD OR “harmful drinking” OR “alcohol misuse” OR “heavy drinking” OR “binge drinking” OR “alcohol use disorder” OR “alcohol abuse” OR “problem drinking” OR “alcohol dependence” OR alcoholism OR alcoholic OR “substance-related disorders” OR “drug abuse” OR “Drug Addiction” OR “Drug Dependence” OR “Drug Use Disorders” OR “Prescription Drug Abuse” OR “Substance Abuse” OR “Substance Addiction” OR “Substance Dependence” OR “Substance Use Disorder” OR “Intoxication” OR “drug misuse” OR “substance use” OR “substance misuse” OR “opiate use” OR “opiate misuse” OR “opiate abuse” OR “opiate Addiction” OR “opiate Dependence” OR “cocaine use” OR “cocaine misuse” OR “cocaine abuse” OR “cocaine Addiction” OR “cocaine Dependence” OR “marijuana use” OR “marijuana misuse” OR “marijuana abuse” OR “marijuana Addiction” OR “stimulant use” OR “stimulant misuse” OR “stimulant abuse” OR “stimulant Addiction” OR “stimulant Dependence” OR “heroin use” OR “heroin misuse” OR “heroin abuse” OR “heroin Addiction” OR “heroin Dependence”

1. *AND “Community-based” OR*

“community health service” OR “community mental health” OR “community care” OR “primary care” OR "community health" OR rural OR local OR integrated OR “community treatment” OR “community intervention”

1. *AND Intervention OR*

prevention OR prevent OR treatment OR treat OR care OR service OR initiative OR counseling OR therapy OR program OR outpatient OR “therapeutic intervention” OR rehabilitation OR “psychiatric rehabilitation” OR “self-help” OR “mutual support” OR “peer support” OR psychoeducation OR “brief intervention” OR “psychosocial intervention” OR “motivational interviewing” OR “relapse prevention” OR support OR “harm reduction” OR “psychological treatment”

1. *“Low-and middle-income country” OR*

"Low middle income country" or LMIC or Albania or Ecuador or Montenegro or Algeria or Fiji or Namibia or “American Samoa” or Gabon or Palau or Angola or Georgia or Panama or Argentina or Grenada or Paraguay or Azerbaijan or Guyana or Peru or Belarus or Iran or Romania or Belize or Iraq or “Russian Federation” or Russia or “Bosnia and Herzegovina” or Bosnia or Jamaica or Serbia or Botswana or Jordan or “South Africa” or Brazil or Kazakhstan or “St. Lucia” or Bulgaria or Lebanon or “St. Vincent and the Grenadines” or China or Libya or Suriname or Colombia or Macedonia or Thailand or “Costa Rica” or Malaysia or Turkey or Cuba or Maldives or Turkmenistan or Dominica or “Marshall Islands” or Tuvalu or “Dominican Republic” or Mauritius or Venezuela or “Equatorial Guinea” or Mexico or Armenia or Kiribati or “Solomon Islands” or Bangladesh or Kosovo or “Sri Lanka” or Bhutan or “Kyrgyz Republic” or Sudan or Bolivia or Lao or Swaziland or “Cabo Verde” or “Cape Verde” or Lesotho or “Syrian Arab Republic” or Syria or Cambodia or Mauritania or Tajikistan or Cameroon or “Micronesia, Fed. Sts.” or Timor-Leste or “Congo, Republic” or Congo or Moldova or Tonga or “Côte d´Ivoire” or Mongolia or Tunisia or Djibouti or Morocco or Ukraine or Egypt or “Egypt Arab Republic” or Myanmar or Uzbekistan or “El Salvador” or Nicaragua or Vanuatu or Ghana or Nigeria or Vietnam or Guatemala or Pakistan or “West Bank” and Gaza or Honduras or “Papua New Guinea” or Yemen or “Yemen Republic” or India or Philippines or Zambia or Indonesia or Samoa or Kenya or “São Tomé and Principe” or Afghanistan or Guinea or Rwanda or Benin or Guinea-Bissau or Senegal or “Burkina Faso” or Haiti or “Sierra Leone” or Burundi or Korea or Somalia or “Central African Republic” or Liberia or “South Sudan” or Chad or Madagascar or Tanzania or Comoros or Malawi or Togo or Congo or Mali or Uganda or Eritrea or Mozambique or Zimbabwe or Ethiopia or Nepal or Gambia or Niger

Limiters: Publication date 2008 to 2019, English language, and academic journal publications (when applicable, peer-reviewed publications).

Appendix S2: Inclusion articles screener sheet

|  | **Yes** | **Specification** |
| --- | --- | --- |
| **Population** | | |
| +16 years under 85 |  |  |
| SUD present (assessed) |  |  |
| SUD present (self-reported) |  |  |
| SUD present (unspecified) |  |  |
| **Community-based Intervention** | | |
| Community settings (specify) |  |  |
| primary care setting |  |  |
| general hospital |  |  |
| out-patient service |  |  |
| mental health center (including day care center) |  |  |
| Self-help group setting |  |  |
| Social services |  |  |
| Vocational support |  |  |
| Other community-based care setting (specify) |  |  |
| **Intervention type** | | |
| Assertive community treatment |  |  |
| cognitive behavioral therapy (CBT) based |  |  |
| brief (indicated prevention) intervention |  |  |
| Psychoeducation |  |  |
| Relapse prevention |  |  |
| Motivational interviewing |  |  |
| Other psychosocial or behavioural intervention (specify) |  |  |
| **Study design** | | |
| Descriptive case study |  |  |
| Evaluation study |  |  |
| Qualitative study (specify) |  |  |
| Mixed method study (specify) |  |  |
| Quantitative study (specify) |  |  |
| **Publication type and date** | | |
| English language |  |  |
| Peer-review journal publication |  |  |
| Publication date 2008-2019 |  |  |

Appendix S3: Sample data capture sheet

| **Reference** |  |
| --- | --- |
| **Abstract** |  |
| **Study design/methods** |  |
| **Target population age** |  |
| **Target population gender** |  |
| **Target SUD** |  |
| **Other target disorder** |  |
| **Identification method** |  |
| **Intervention model/name** |  |
| **Study/intervention objectives** |  |
| **Intervention components** |  |
| **Cultural adaptations made** |  |
| **Duration of intervention** |  |
| **Persons delivering the intervention** |  |
| **Level of implementation (local, regional, national)** |  |
| **Delivery/implementation method** |  |
| **Intervention setting (geographical location/s and delivering-organization)** |  |
| **Measures** |  |
| **Reported outcomes** |  |
| **Policy barriers or facilitators discussed** |  |
| **Resource barriers discussed** |  |
| **Resource facilitators discussed** |  |
| **Social/cultural barriers discussed** |  |
| **Social/cultural facilitators discussed** |  |
| **Other barriers or study limitations discussed** |  |
| **Other intervention facilitators discussed** |  |

| Appendix S4: PRISMA checklist | | | |
| --- | --- | --- | --- |
| **Section/topic** | **#** | **Checklist item** | **Reported on page #** |
| **TITLE** | | |  |
| Title | 1 | Identify the report as a systematic review, meta-analysis, or both. | 2 |
| **ABSTRACT** | | |  |
| Structured summary | 2 | Provide a structured summary including, as applicable: background; objectives; data sources; study eligibility criteria, participants, and interventions; study appraisal and synthesis methods; results; limitations; conclusions and implications of key findings; systematic review registration number. | 2 |
| **INTRODUCTION** | | |  |
| Rationale | 3 | Describe the rationale for the review in the context of what is already known. | 3-8 |
| Objectives | 4 | Provide an explicit statement of questions being addressed with reference to participants, interventions, comparisons, outcomes, and study design (PICOS). | 8 |
| **METHODS** | | |  |
| Protocol and registration | 5 | Indicate if a review protocol exists, if and where it can be accessed (e.g., Web address), and, if available, provide registration information including registration number. | N/A |
| Eligibility criteria | 6 | Specify study characteristics (e.g., PICOS, length of follow-up) and report characteristics (e.g., years considered, language, publication status) used as criteria for eligibility, giving rationale. | 9-10 |
| Information sources | 7 | Describe all information sources (e.g., databases with dates of coverage, contact with study authors to identify additional studies) in the search and date last searched. | 11 |
| Search | 8 | Present full electronic search strategy for at least one database, including any limits used, such that it could be repeated. | Appendix 1 |
| Study selection | 9 | State the process for selecting studies (i.e., screening, eligibility, included in systematic review, and, if applicable, included in the meta-analysis). | 10-11, Appendix 2 |
| Data collection process | 10 | Describe method of data extraction from reports (e.g., piloted forms, independently, in duplicate) and any processes for obtaining and confirming data from investigators. | 10-11 |
| Data items | 11 | List and define all variables for which data were sought (e.g., PICOS, funding sources) and any assumptions and simplifications made. | 9-10, Table 1, Appendix 3 |
| Risk of bias in individual studies | 12 | Describe methods used for assessing risk of bias of individual studies (including specification of whether this was done at the study or outcome level), and how this information is to be used in any data synthesis. | N/A |
| Summary measures | 13 | State the principal summary measures (e.g., risk ratio, difference in means). | N/A |
| Synthesis of results | 14 | Describe the methods of handling data and combining results of studies, if done, including measures of consistency (e.g., I^2^) for each meta-analysis. | N/A |

| **Section/topic** | **#** | **Checklist item** | **Reported on page #** |
| --- | --- | --- | --- |
| Risk of bias across studies | 15 | Specify any assessment of risk of bias that may affect the cumulative evidence (e.g., publication bias, selective reporting within studies). | 23 |
| Additional analyses | 16 | Describe methods of additional analyses (e.g., sensitivity or subgroup analyses, meta-regression), if done, indicating which were pre-specified. | N/A |
| **RESULTS** | | |  |
| Study selection | 17 | Give numbers of studies screened, assessed for eligibility, and included in the review, with reasons for exclusions at each stage, ideally with a flow diagram. | 12 |
| Study characteristics | 18 | For each study, present characteristics for which data were extracted (e.g., study size, PICOS, follow-up period) and provide the citations. | 13-15, Table 3a & 3b |
| Risk of bias within studies | 19 | Present data on risk of bias of each study and, if available, any outcome level assessment (see item 12). | N/A |
| Results of individual studies | 20 | For all outcomes considered (benefits or harms), present, for each study: (a) simple summary data for each intervention group (b) effect estimates and confidence intervals, ideally with a forest plot. | N/A (Narrative summary of findings in table 3b) |
| Synthesis of results | 21 | Present results of each meta-analysis done, including confidence intervals and measures of consistency. | N/A |
| Risk of bias across studies | 22 | Present results of any assessment of risk of bias across studies (see Item 15). | N/A |
| Additional analysis | 23 | Give results of additional analyses, if done (e.g., sensitivity or subgroup analyses, meta-regression [see Item 16]). | N/A |
| **DISCUSSION** | | |  |
| Summary of evidence | 24 | Summarize the main findings including the strength of evidence for each main outcome; consider their relevance to key groups (e.g., healthcare providers, users, and policy makers). | 19-23 |
| Limitations | 25 | Discuss limitations at study and outcome level (e.g., risk of bias), and at review-level (e.g., incomplete retrieval of identified research, reporting bias). | 23 |
| Conclusions | 26 | Provide a general interpretation of the results in the context of other evidence, and implications for future research. | 23-24 |
| **FUNDING** | | |  |
| Funding | 27 | Describe sources of funding for the systematic review and other support (e.g., supply of data); role of funders for the systematic review. | 24 |

*From:*  Moher D, Liberati A, Tetzlaff J, Altman DG, The PRISMA Group (2009). Preferred Reporting Items for Systematic Reviews and Meta-Analyses: The PRISMA Statement. PLoS Med 6(7): e1000097. doi:10.1371/journal.pmed1000097

For more information, visit: **www.prisma-statement.org**.
